# Long-term health-related quality of life in patients treated with subcutaneous C1-inhibitor replacement therapy for the prevention of hereditary angioedema attacks: findings from the COMPACT open-label extension study

**DOI:** 10.1186/s13023-020-01658-4

**Published:** 2021-02-15

**Authors:** William R. Lumry, Bruce Zuraw, Marco Cicardi, Timothy Craig, John Anderson, Aleena Banerji, Jonathan A. Bernstein, Teresa Caballero, Henriette Farkas, Richard G. Gower, Paul K. Keith, Donald S. Levy, H. Henry Li, Markus Magerl, Michael Manning, Marc A. Riedl, John-Philip Lawo, Subhransu Prusty, Thomas Machnig, Hilary Longhurst, on behalf of the COMPACT Investigators

**Affiliations:** 1 AARA Research Center, 10100 N. Central Expressway, Suite 100, Dallas, TX 75231 USA; 2 Department of Medicine, UC San Diego, La Jolla, CA USA; 3 University of Milan, Milan, Italy; 4 Penn State University College of Medicine, Hershey, PA USA; 5 Clinical Research Center of Alabama, Birmingham, AL USA; 6 Massachusetts General Hospital, Boston, MA USA; 7 University of Cincinnati College of Medicine and Bernstein Clinical Research Center, LLC, Cincinnati, OH USA; 8 Allergy Department, Hospital La Paz Institute for Health Research (IdiPaz), Biomedical Research Network On Rare Diseases (CIBERER U754), Madrid, Spain; 9 Hungarian Angioedema Reference Center, 3Rd Department of Internal Medicine, Semmelweis University, Budapest, Hungary; 10 Marycliff Clinical Research, Spokane, WA USA; 11 McMaster University, Hamilton, ON USA; 12 UC Irvine, Orange, CA USA; 13 Institute for Asthma and Allergy, Chevy Chase, MD USA; 14 Department of Dermatology and Allergy, Charité, Universitätsmedizin Berlin, Berlin, Germany; 15 Medical Research of Arizona, Scottsdale, AZ USA; 16 Division of Rheumatology, Allergy and Immunology, University of California, San Diego, La Jolla, CA USA; 17 CSL Behring GmbH, Emil-von-Behring-Strasse 76, Marburg, Germany; 18 University College Hospital, London, UK; 19 Auckland District Health Board, Auckland, New Zealand

**Keywords:** C1-inhibitor protein, Hereditary angioedema, Patient-reported outcomes, Health-related quality of life, Productivity, Subcutaneous, HAEGARDA, Anxiety, Depression

## Abstract

**Background:**

Long-term prophylaxis with subcutaneous C1-inhibitor (C1-INH[SC]; HAEGARDA, CSL Behring) in patients with hereditary angioedema (HAE) due to C1-INH deficiency (C1-INH-HAE) was evaluated in an open-label extension follow-up study to the international, double-blind, placebo-controlled COMPACT study. The current analysis evaluated patient-reported health-related quality of life (HRQoL) data from 126 patients in the open-label extension study randomized to treatment with C1-INH(SC) 40 IU/kg (n = 63) or 60 IU/kg (n = 63) twice weekly for 52 weeks. HRQoL was evaluated at the beginning of the open-label study and at various time points using the European Quality of Life-5 Dimensions Questionnaire (EQ-5D), the Hospital Anxiety and Depression Scale (HADS), the Work Productivity and Activity Impairment Questionnaire (WPAI), and the Treatment Satisfaction Questionnaire for Medication. The disease-specific Angioedema Quality of Life Questionnaire (AE-QoL) and HAE quality of life questionnaire (HAE-QoL) instruments were administered in a subset of patients. Statistical significance was determined by change-from-baseline 95% confidence intervals (CIs) excluding zero. No adjustment for multiplicity was done.

**Results:**

Mean baseline EQ-5D scores (Health State Value, 0.90; Visual Analog Scale, 81.32) were slightly higher (better) than United States population norms (0.825, 80.0, respectively) and mean HADS anxiety (5.48) and depression (2.88) scores were within “normal” range (0–7). Yet, patients using C1-INH(SC) 60 IU/kg demonstrated significant improvement from baseline to end-of-study on the EQ-5D Health State Value (mean change [95% CI], 0.07 [0.01, 0.12] and Visual Analog Scale (7.45 [3.29, 11.62]). In the C1-INH(SC) 60 IU/kg group, there were significant improvements in the HADS anxiety scale (mean change [95% CI], − 1.23 [− 2.08, − 0.38]), HADS depression scale (− 0.95 [− 1.57, − 0.34]), and WPAI-assessed presenteeism (mean change [95% CI], − 23.33% [− 34.86, − 11.81]), work productivity loss (− 26.68% [− 39.92, − 13.44]), and activity impairment (− 16.14% [− 26.36, − 5.91]). Clinically important improvements were achieved in ≥ 25% of patients for all domains except WPAI-assessed absenteeism (which was very low at baseline). Mean AE-QoL total score by visit ranged from 13.39 to 17.89 (scale 0–100; lower scores = less impairment). Mean HAE-QoL global scores at each visit (115.7–122.3) were close to the maximum (best) possible score of 135.

**Conclusions:**

Long-term C1-INH(SC) replacement therapy in patients with C1-INH-HAE leads to significant and sustained improvements in multiple measures of HRQoL.

*Trial registration* A Study to Evaluate the Long-term Clinical Safety and Efficacy of Subcutaneously Administered C1-esterase Inhibitor in the Prevention of Hereditary Angioedema, NCT02316353. Registered December 12, 2014, https://clinicaltrials.gov/ct2/show/NCT02316353.

## Background

Hereditary angioedema (HAE) due to C1-inhibitor (C1-INH) deficiency (C1-INH-HAE) is a rare, chronic, and potentially debilitating disease. Patients with C1-INH-HAE are prone to recurring and generally unpredictable episodes of subcutaneous or submucosal edema that may affect various organ systems, most commonly the skin, upper airways, and gastrointestinal tract [1]. HAE attacks can be painful and can interfere with personal functioning, often leading to absenteeism from work and school [2–6].

Beyond the direct physical burdens of C1-INH-HAE, it is common for patients to experience anxiety between HAE attacks due to factors such as the unpredictability of attacks, fear of pain associated with the swelling, and anxieties about potentially fatal laryngeal swelling [7–9]. Not surprisingly, a high burden of depression has also been reported in patients with C1-INH-HAE [9, 10]. The negative impact of HAE on health-related quality of life (HRQoL) has been well documented in a number of studies [2, 9–20], and improving HRQoL has become an increasing focus of HAE management guidelines [21].

Subcutaneous administration of C1-INH was first described in a case report published in 2015 [22]. The efficacy, safety, and HRQoL benefits of the marketed formulation of subcutaneous C1-INH (C1-INH[SC]; HAEGARDA, CSL Behring) as prophylaxis in patients with hereditary angioedema were formally studied in the large COMPACT trial program [23–25]. In the pivotal phase 3, placebo-controlled, crossover design COMPACT study, prophylaxis with twice weekly C1-INH(SC) over a 4-month period reduced the mean number of HAE attacks per month relative to placebo by − 2.42 (40 IU/kg) (95% confidence interval [CI], − 3.38 to − 1.46) and − 3.51 (60 IU/kg) (95% CI, − 4.21 to − 2.81), which corresponded to median percent reductions of 89% and 95%, respectively [24]. Prophylaxis with C1-INH(SC) was associated with better general health, less anxiety, less work presenteeism (health-related productivity impairment at work), less work productivity loss, and less activity impairment (both doses combined) compared to placebo [25]. For each HRQoL outcome, a greater proportion of patients had a clinically meaningful improvement during C1-INH(SC) treatment than during placebo treatment.

The long-term safety and efficacy of C1-INH(SC) were investigated in a recently completed open-label follow-on study to the double-blind COMPACT study. The open-label study included 126 patients with C1-INH-HAE treated with C1-INH(SC) 40 or 60 IU/kg twice weekly for a mean (SD) of 76 (40) weeks and longer than 2 years in more than one-third of patients. Safety and efficacy findings from the open-label extension have been described elsewhere [26]. This report presents patient-reported HRQoL outcome data from the long-term COMPACT extension study.

## Methods

### Study design

This was an open-label, international (11 countries), multicenter (32 centers) parallel-arm extension study (NCT02316353) performed as a follow-on to the double-blind, crossover design, phase 3 COMPACT study [24]. The COMPACT study was conducted in accordance with the standards of Good Clinical Practice as defined by the International Council for Harmonization of Technical Requirements for Registration of Pharmaceuticals for Human Use, ethical principles that have their origin in the Declaration of Helsinki, and applicable national and local regulations. The study protocol and any amendments were approved by independent ethics committees or institutional review boards at all participating centers prior to study commencement.

Study design details and clinical findings of the open-label extension study have been published previously [26]. To summarize, the study enrolled 126 patients ≥ 6 years of age with type 1 or type 2 C1-INH-HAE between December 2014 and May 2016. Patients from the double-blind phase 3 COMPACT study, as well as study treatment-naïve patients were eligible to enroll. Patient eligibility required a “baseline” HAE attack history of ≥ 4 attacks within 2 consecutive months; this reflected the period just prior to enrollment in the double-blind COMPACT study or, for naïve patients, prior to enrollment in the open-label extension. Patients were randomized 1:1 to receive open-label treatment with C1-INH(SC) 40 or 60 IU/kg twice weekly for 52 weeks, separated into two treatment periods (24 weeks and 28 weeks, respectively), during which conditional up-titration of dosing was allowed, to optimize prophylaxis. A country-specific protocol amendment gave patients at sites in the United States (US) the option of continuing open-label treatment for an additional 88-week extension period beyond the 52 weeks.

### Patient- and investigator-reported outcomes

**Fig. 1:**
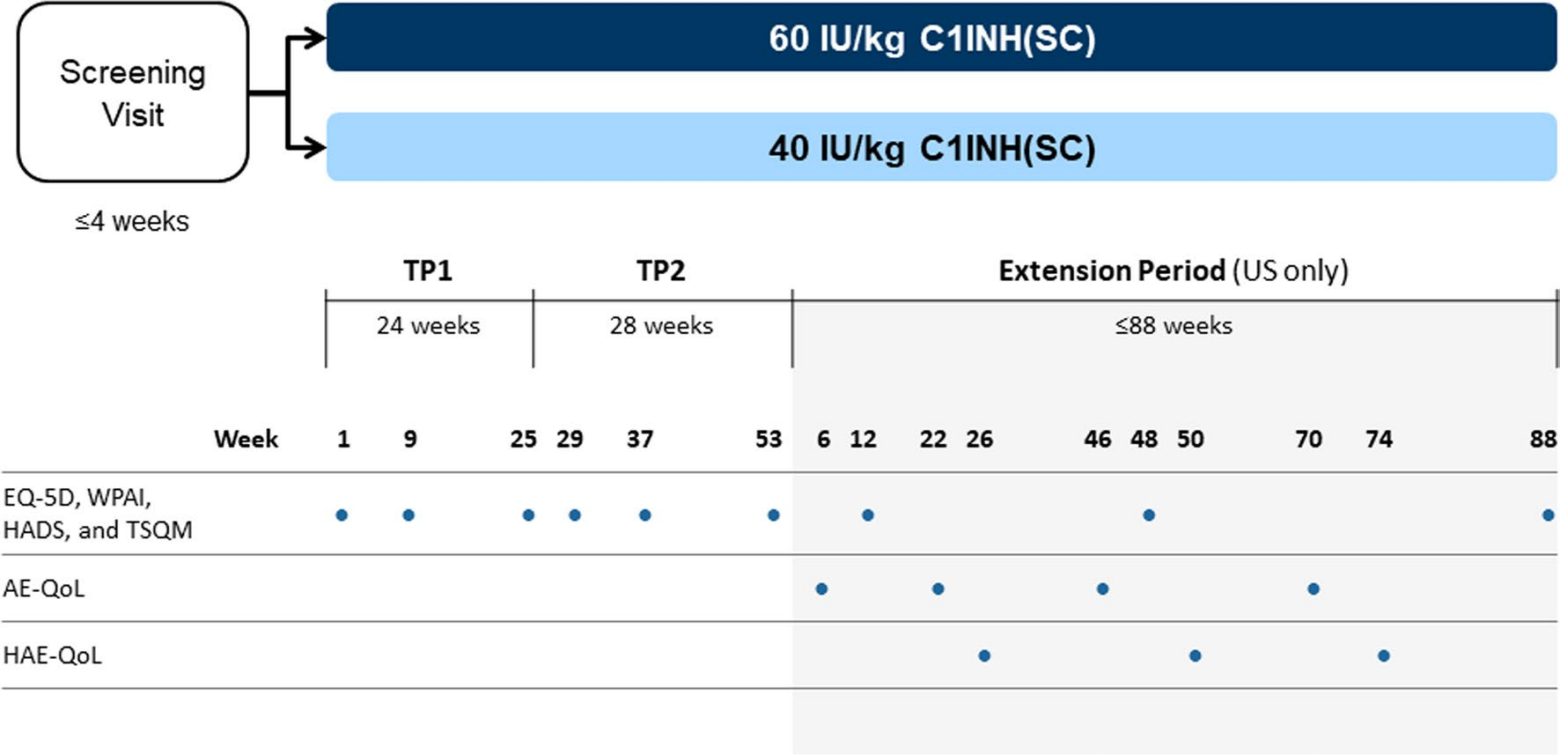
COMPACT open-label study design and timing of HRQoL assessments.* AE-QoL* Angioedema Quality of Life Questionnaire, *C1-INH(SC)* subcutaneous C1-inhibitor, *EQ-5D* European Quality of Life-5 Dimensions Questionnaire, *HADS* Hospital Anxiety and Depression Scale,* HAE-QoL* Hereditary Angioedema Quality of Life Questionnaire,* HRQoL* health-related quality of life, *TSQM* Treatment Satisfaction Questionnaire for Medication, *TP* treatment period, *US* United States, *WPAI* Work Productivity and Activity Impairment Questionnaire

As exploratory outcomes during the open-label study, HRQoL measures were self-administered by patients at baseline and at various times throughout the study using several commonly used HRQoL instruments (Fig. 1): the European Quality of Life-5 Dimensions Questionnaire 3-level version (EQ-5D-3L) [27] as a measure of general health and health status; the Hospital Anxiety and Depression Scale (HADS) [28] to assess anxiety and depression; the Work Productivity and Activity Impairment Questionnaire (WPAI) [29] to evaluate health-related work productivity and activity impairment; and the Treatment Satisfaction Questionnaire for Medication (TSQM) [30] to measure patients’ satisfaction with their treatment.

During the additional 88-week extension period, HAE-related HRQoL was also assessed using new disease-specific instruments, the angioedema quality of life (AE-QoL) and the hereditary angioedema quality of life (HAE-QoL), which became available during the course of the study. The AE-QoL questionnaire, published in 2012 [31], is the first validated angioedema-specific HRQoL instrument developed to assess HRQoL in patients with recurrent angioedema, including both chronic urticaria and HAE. Patients with both conditions were included in the AE-QoL item generation and studies for instrument validation. The AE-QoL is a self-administered questionnaire consisting of 17 questions grouped into four domains (functioning, fatigue/mood, fears/shame, and food). Responses are based on a 5-point Likert scale of “never” to “very often” with a recall period of 4 weeks. Scores are transformed to a linear scale ranging from 0 to 100 with higher scores representing greater HRQoL impairment. The AE-QoL is available in multiple languages.

The HAE-QoL is a questionnaire specifically designed to study the impact of C1-INH-HAE on adult patients’ HRQoL [32]. The HAE-QoL, available in multiple languages, includes 25 questions assigned to one of seven relevant HRQoL domains (physical functioning and health, disease-related stigma, emotional role and social functioning, concern about offspring, perceived control over illness, mental health, and treatment difficulties) and has a recall period of 6 months. Higher scores are associated with less HRQoL impairment.

### Data collection and analysis

HRQoL data were collected using an electronic diary completed by the subjects at the study center. The analysis population consisted of all patients in the open-label intent-to-treat population who provided at least 1 HRQoL assessment. Regardless of dose titration, patients were analyzed in the treatment group to which they were randomized. All HRQoL score values were summarized by visit and treatment; missing values for the HRQoL assessments were not imputed. “Baseline” for the European Quality of Life-5 Dimensions Questionnaire (EQ-5D), HADS, WPAI, and TSQM was visit 1 (week 1) of the open-label study.

For the EQ-5D, HADS, WPAI, and TSQM, total scores for each domain or each questionnaire (as applicable) were evaluated for treatment effect as follows: intra-subject differences of C1-INH(SC) 60 IU/kg versus baseline and C1-INH(SC) 40 IU/kg versus baseline were calculated for the final visit (week 53 or extension period week 88 for subjects who participated in the extension period). Two-sided 95% CIs for the mean and median differences were produced. Statistically significant differences were concluded if the respective CI excluded 0, without adjustment for multiplicity. Subjects with one or two questions/items or dimensions (as applicable) missing at the baseline visit or the final visit (week 53 or extension phase week 88) were excluded from this analysis. The minimal clinically important difference (MCID) for each outcome was estimated using the half standard deviation (SD) approach, which has been shown to approximate the threshold of discrimination for a clinically meaningful change or difference in patient-reported outcome scores for patients with chronic diseases [33]. For the AE-QoL and HAE-QoL, mean/median scores were determined for each assessment time point and analyzed descriptively.

## Results

### Study population

The open-label study enrolled and randomized 126 patients to C1-INH(SC) 40 IU/kg (n = 63) or C1-INH(SC) 60 IU/kg (n = 63) (Table 1). All 126 patients were included in the HRQoL population. The mean (SD) age was 40.5 (15.56) years (range, 8–72 years). Three patients were between the ages of 8 and 11 years, and seven subjects were between the ages of 12 and 16 years. A majority of patients were female (60.3%), white (96.0%), and had C1-INH(SC)-HAE type 1 (89.7%).

**Table 1:** Baseline characteristics of patients in COMPACT long-term, open-label study (HRQoL population)

	C1-INH(SC) 40 IU/kg N = 63	C1-INH(SC) 60 IU/kg N = 63	All C1-INH(SC) N = 126
Age, years, mean (SD)^a^	40.8 (14.96)	40.3 (16.26)	40.5 (15.56)
Range	8–67	10–72	8–72
Female, n (%)	40 (63.5)	36 (57.1)	76 (60.3)
Mean body weight, kg (SD)	86.1 (23.27)	84.26 (24.24)	85.2 (23.68)
Mean BMI, kg/m^2^ (SD)	29.6 (6.92)	28.8 (7.56)	29.2 (7.23)
Race, n (%)			
White	60 (95.2)	61 (96.8)	121 (96.0)
Black or African American	1 (1.6)	1 (1.6)	2 (1.6)
Asian	0	1 (1.6)	1 (0.8)
Other	2 (3.2)	0	2 (1.6)
HAE history, n (%)			
C1-INH(SC)-HAE type I	55 (87.3)	58 (92.1)	113 (89.7)
C1-INH(SC)-HAE type II	8 (12.7)	5 (7.9)	13 (10.3)
Mean (SD) number HAE attacks in 3 months before screening^b^	12.8 (8.42)	12.7 (10.23)	12.8 (9.33)
Prior use of prophylaxis (preceding 3 months), n (%)	39 (61.9)	40 (63.5)	79 (62.7)
Prior prophylaxis medication			
C1-INH(SC) (COMPACT study)	32 (50.8)	32 (50.8)	64 (50.8)
C1-INH(IV)	6 (9.5)	5 (7.9)	11 (8.7)
Oral prophylaxis (danazol)	1 (1.6)	3 (4.8)	4 (3.2)

*BMI* body mass index, *HAE* hereditary angioedema, *SD* standard deviation,* C1-INH(SC)* subcutaneous C1-inhibitor,* HRQoL* health-related quality of life,* C1-INH(IV)* intravenous C1-inhibitor

^a^Ten patients were < 18 years old (range: 8–16 years, with three patients < 12 years old) and 10 patients were ≥ 65 years old (range: 65–72 years)

^b^For C1-INH(SC)-naïve subjects and C1-INH(SC)-interrupted subjects, “screening” was the first visit of the open-label study; for C1-INH(SC)-continuation subjects, the screening visit was prior to entry into the double-blind COMPACT study

Half of the patients in the study (n = 64, 50.8%) were participants in the double-blind COMPACT study, thus were using C1-INH(SC) prophylaxis prior to continuing into the open-label study. Another one-third of the population (n = 44; 34.9%) was using other HAE prophylaxis (intravenous C1-INH [C1-INH{IV)] or oral danazol) within a 3-month period prior to the open-label study (Table 1). Only 19 (15.1%) patients were not using any HAE prophylaxis within 3 months prior to the open-label study.

### Patient disposition and main efficacy and safety outcomes

Open-label treatment periods 1 and 2 (total of 52 weeks) were completed by 55 (87.3%) and 55 (87.3%) of patients in the C1-INH(SC) 40 and 60 IU/kg treatment groups, respectively. The additional 88-week extension period in the US was completed by 22 of 22 (100%) patients in the 40 IU/kg group and 23 of 24 (95.8%) patients in the 60 IU/kg group.

The median attack rates during the open-label study in the C1-INH(SC) 60 IU/kg and 40 IU/kg groups, respectively, were 0.09 and 0.11 attacks per month (corresponding to annualized rates of 1.0 and 1.3 attacks per year, respectively). No HAE attacks or mild attacks only were reported by 36 (57.1%) patients in the C1-INH(SC) 60 IU/kg group and 31 (49.2%) patients in the C1-INH(SC) 40 IU/kg group. Among 23 patients who used the approved dose of 60 IU/kg for more than 2 years, 19 (82.6%) were completely attack-free during study months 25–30 and 20 (87.0%) reported no rescue medication use during study months 25–30 [26].

The incidence of adverse events (AEs) was low and similar in both dose groups (8.5 and 11.3 AEs per patient, year of exposure in the 60 and 40 IU/kg treatment groups, respectively). Mild injection site reactions were the most commonly reported AEs, and there were no serious events considered related to C1-INH(SC) treatment [26].

### European quality of life-5 dimensions Questionnaire

**Fig. 2:**
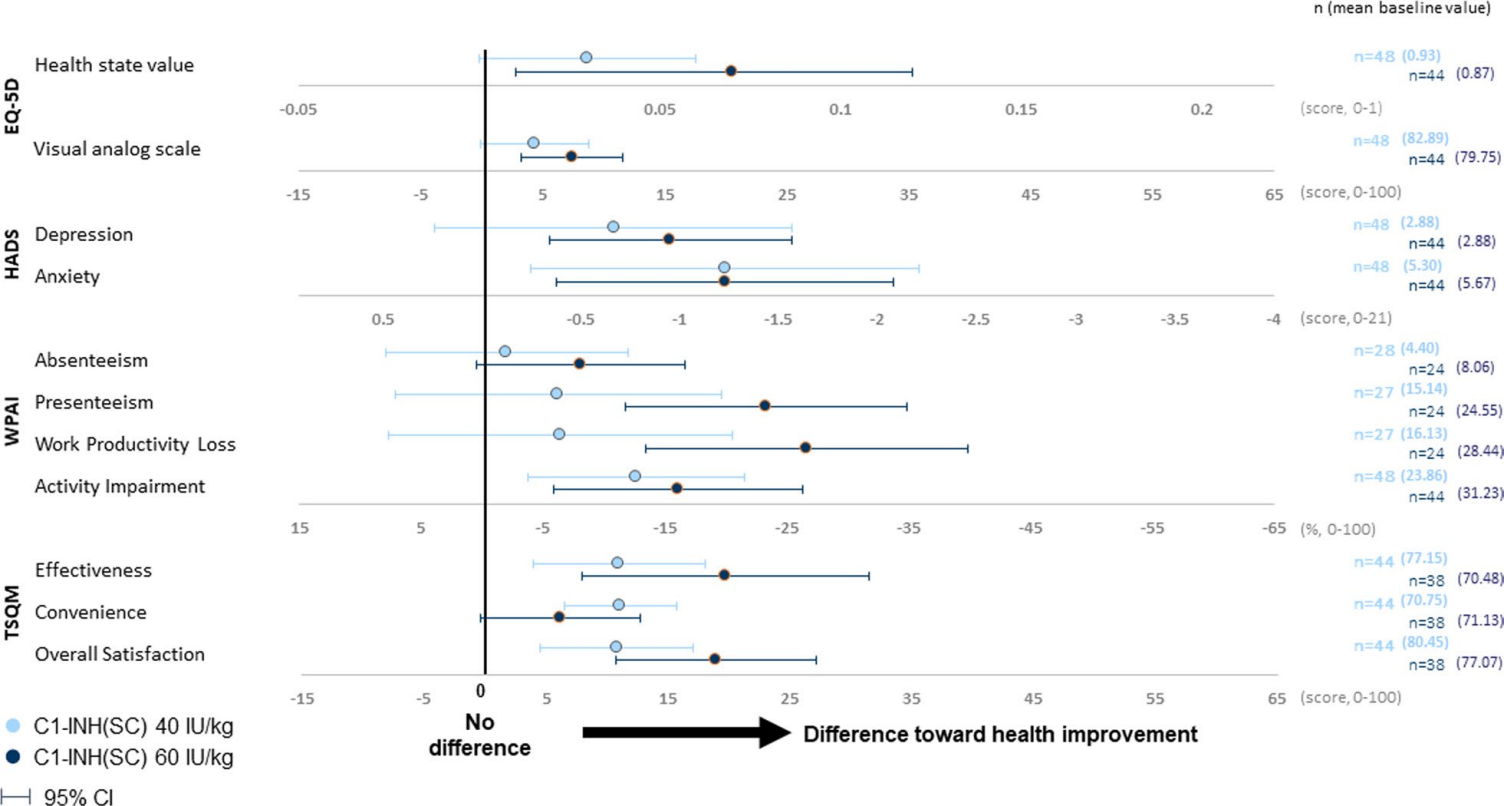
Mean intra-subject change from baseline (95% CI) in patient-reported outcomes, baseline^a^ to end of study^b^. *Note* Mean and 95% CI values can be found in Additional file 1. ^a^Baseline was visit 1 (week 1) of the open-label study. ^b^Final visit (week 53 or extension period week 88 for subjects who participated in the extension period). *C1-INH(SC)* subcutaneous C1-inhibitor, *CI* confidence interval, *EQ-5D* European Quality of Life-5 Dimensions Questionnaire, *HADS* Hospital Anxiety and Depression Scale, *TSQM* Treatment Satisfaction Questionnaire for Medication, *WPAI* Work Productivity and Activity Impairment Questionnaire, *CI* confidence interval

At the open-label study baseline, mean EQ-5D Health State Value and Visual Analog Scale (VAS) scores in the study population were high and slightly better than US population norms (Table 2), suggesting good self-reported quality of life in the study subjects, all of whom had immediate access to treatment for acute attacks and most of whom had been using some form of prophylaxis already prior to study entry. Yet, from baseline to the final study visit (week 53 or extension period week 88 for patients who participated in the additional extension period in the US), patients in the C1-INH(SC) 60 IU/kg treatment group demonstrated a further and significant improvement on both the EQ-5D Health State Value (mean change [95% CI], 0.07 [0.01, 0.12] and the EQ-5D VAS (mean change [95% CI], 7.45 [3.29, 11.62]) (Fig. 2). In the C1-INH(SC) 40 IU/kg treatment group, mean changes from baseline demonstrated a trend for improvement but did not reach statistical significance (EQ-5D Health State Value, mean [95% CI] change from baseline 0.03 [0, 0.06]; VAS, 4.33 [− 0.13, 8.80]) (Fig. 2).

**Table 2:** HRQoL assessments (EQ-5D, HADS, WPAI, TSQM) at baseline and end-of-study, both C1-INH(SC) doses combined (N = 126)

HRQoL assessment	Scoring and interpretation	Baseline		End of study (week 53 or extension week 88)		Intra-subject change from baseline^a^	
		n	Mean (SD)	n	Mean (SD)	n	Mean (SD)
*EQ-5D*							
Health state value	Health state value: scored from 0 (dead) to 1 (full health); population norm in the US = 0.825 [34] VAS: scored from 0 (worst) to 100 (best imaginable health state); population norm in the US = 80.0 [34]	114	0.90 (0.168)	100	0.95 (0.098)	92	0.05 (0.153)
VAS		114	81.32 (17.600)	100	87.72 (13.036)	92	5.83 (14.601)
*HADS*							
Depression	*Both domains:* 0–7 (normal) 8–10 (suggestive of the mood disorder) > 11 (probable presence of the mood disorder) Maximum (worst) score possible per domain = 21	114	2.88 (2.930)	100	1.91 (2.629)	92	− 0.80 (2.641)
Anxiety		114	5.48 (3.863)	100	4.11 (3.533)	92	− 1.23 (3.089)
*WPAI*							
Absenteeism (% work time missed)^b^	*All domains* Higher % = greater impairment (maximum score = 100%)	69	6.15 (17.056)	68	2.67 (11.670)	52	− 4.89 (23.296)
Presenteeism (% impairment while working)^b^		68	19.71 (26.653)	68	8.24 (18.604)	51	− 14.31 (31.765)
Work productivity loss (% overall work impairment)^b^		68	22.11 (29.018)	68	9.45 (20.766)	51	− 15.97 (34.946)
Activity impairment (% activity (non-work) impairment		114	27.54 (29.436)	100	14.40 (24.917)	92	− 14.35 (32.013)
*TSQM*							
Effectiveness	*Each domain* Scoring range is 0–100; higher scores indicate better outcomes	104	73.88 (25.000)	100	83.67 (27.237)	82	15.04 (29.854)
Convenience		104	70.94 (16.965)	100	80.56 (17.470)	82	8.81 (17.632)
Overall satisfaction		104	78.79 (22.414)	100	92.42 (13.253)	82	14.57 (23.016)

*C1-INH(SC)* subcutaneous C1-inhibitor, *EQ-5D* European Quality of Life-5 Dimensions Questionnaire, *HADS* Hospital Anxiety and Depression Scale, *HRQoL* health-related quality of life, *TSQM* Treatment Satisfaction Questionnaire for Medication, *WPAI* Work Productivity and Activity Impairment Questionnaire,* SD* standard deviation,* VAS* visual analog scale

^a^The Change from Baseline analysis included only patients with both Baseline and End of Study scores, thus the number of patients in this column may be smaller than that shown in the End of Study column as a consequence of missing baseline data

^b^Assessment completed only by employed patients

### Hospital Anxiety and Depression Scale (HADS)

#### HADS depression domain

At baseline, the mean HADS depression domain score was suggestive of a low overall burden of depression in this population (2.88 [0 = best possible score; 21 = worst possible score]) (Table 2). Yet, a significant improvement (score decrease) from baseline to the end-of-study visit was observed in the C1-INH(SC) 60 IU/kg treatment group (mean change − 0.95, 95% CI − 1.57, − 0.34]), while a non-significant decrease in mean depression score was noted in the C1-INH(SC) 40 IU/kg group from baseline to end of study (mean change, − 0.67; 95% CI − 1.57, 0.24) (Fig. 2).

#### HADS anxiety domain

At baseline, the mean HADS anxiety score was 5.48, which is within the scoring range for what is considered “normal” for this domain (0–7). The mean score at end of study was 4.11 in both C1-INH(SC) dose groups, representing significant improvements from baseline (Fig. 2). The mean change from baseline was − 1.23 in both the 60 IU/kg group (95% CI − 2.08, − 0.38) and the 40 IU/kg group (95% CI − 2.21, − 0.25).

### Work Productivity and Activity Impairment (WPAI) Questionnaire

Baseline values for presenteeism (health-related productivity impairment at work), work productivity loss (absenteeism plus presenteeism), and activity impairment (% of non-work activity impairment) indicated moderate levels of impairment for these outcomes (19.71%, 22.11%, and 27.54%, respectively) (Table 2). In the C1-INH(SC) 60 IU/kg group, there were significant improvements from baseline in mean values for these three domains including presenteeism (mean change [95% CI], − 23.33% [− 34.86, − 11.81]), work productivity loss (− 26.68% [− 39.92, − 13.44]), and activity impairment (− 16.14% [− 26.36, − 5.91]) (Fig. 2). In the C1-INH(SC) 40 IU/kg group, there was a significant improvement from baseline in activity impairment only (mean change [95% CI], − 12.71 [− 21.63, − 3.79]). No significant changes from baseline for absenteeism, which was low at baseline (6.15%), were seen for either dose group.

### Treatment Satisfaction Questionnaire for Medication

Baseline scores for TSQM effectiveness, convenience, and overall satisfaction were moderately high, ranging from 70.94 to 78.79 on a scale of 0 (worst) to 100 (best) (Table 2). In the C1-INH(SC) 60 IU/kg treatment group, significant improvements from baseline to end of study were observed for the TSQM domains of effectiveness (mean change [95% CI], 19.74 [7.94, 31.54]) and overall satisfaction (18.93 [10.68, 27.18]) (Fig. 2). In the C1-INH(SC) 40 IU/kg group, significant improvements were noted in scores for effectiveness (mean change [95% CI], 10.98 [3.96, 18.01]), convenience (11.11 [6.47, 15.75]), and overall satisfaction (10.80 [4.53, 17.06]).

Mean and median changes from baseline to the end of study for the EQ-5D, HADS, WPAI, and TSQM assessments are presented in Additional file 1. Mean EQ-5D, HADS, WPAI, and TSQM scores by individual study visit for all C1-INH(SC) doses combined are provided in Additional file 2, Additional file 3, Additional file 4, and Additional file 5.

### Minimal clinically important differences

**Fig. 3:**
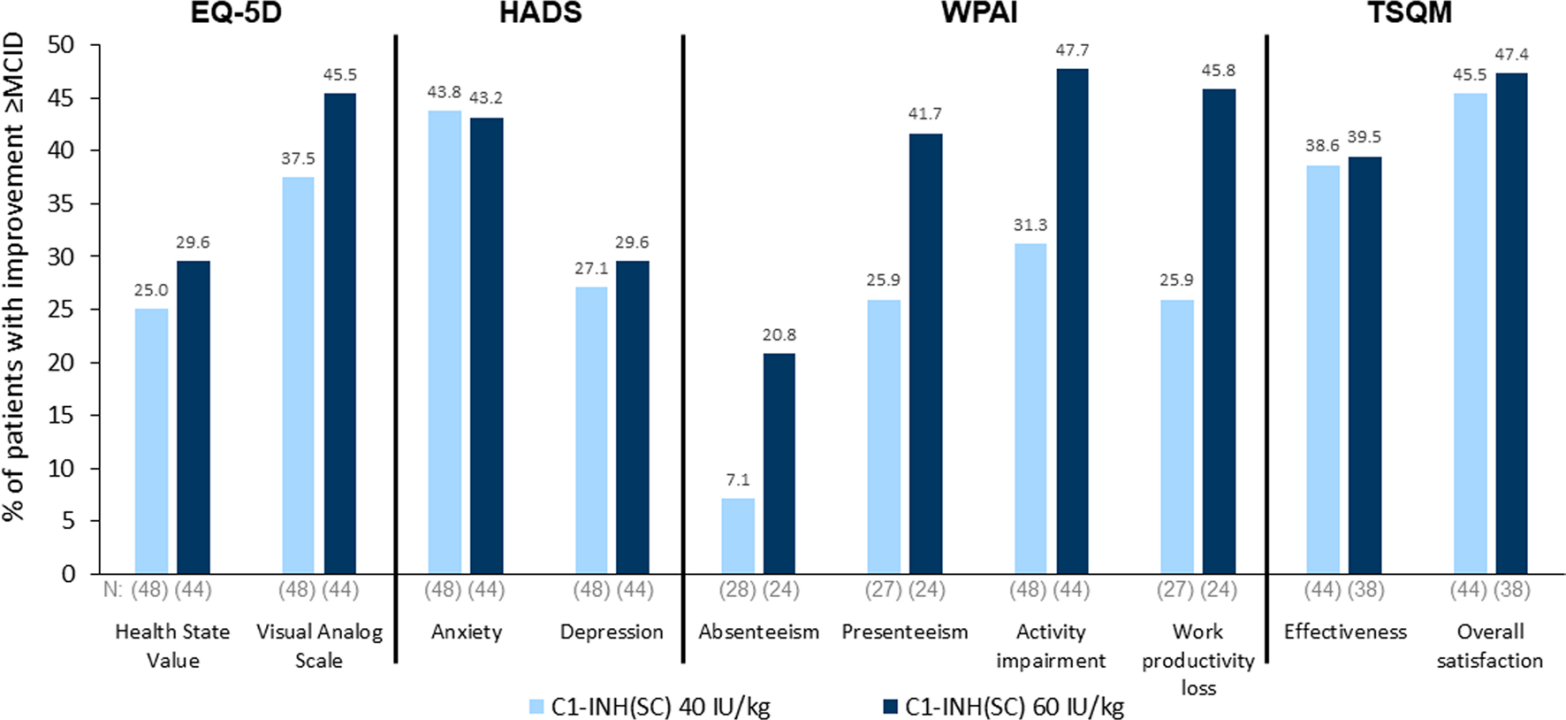
Percentages of patients using C1-INH(SC) who demonstrated minimal clinically important difference (MCID) improvements from baseline to end-of-study. *C1-INH(SC)* subcutaneous C1-inhibitor, *EQ-5D* European Quality of Life-5 Dimensions Questionnaire, *HADS* Hospital Anxiety and Depression Scale, *TSQM* Treatment Satisfaction Questionnaire for Medication, *WPAI* Work Productivity and Activity Impairment Questionnaire

The proportions of patients with changes from baseline in EQ-5D, HADS, WPAI, and TSQM scores meeting MCID criteria while using C1-INH(SC) are shown in Fig. 3. Excluding absenteeism (which was very low at baseline), 25% or more of patients achieved MCID improvements from baseline for all assessments. For a number of outcomes, almost half of patients using C1-INH(SC) achieved MCID improvements from baseline, including EQ-5D VAS (60 IU/kg group); HADS anxiety (both doses); WPAI presenteeism, activity impairment, and work productivity loss (60 IU/kg); and TSQM overall satisfaction (both doses). For absenteeism, 7.1% (40 IU/kg) and 20.8% (60 IU/kg) of patients had MCID improvements from baseline.

### AE-QoL

**Fig. 4:**
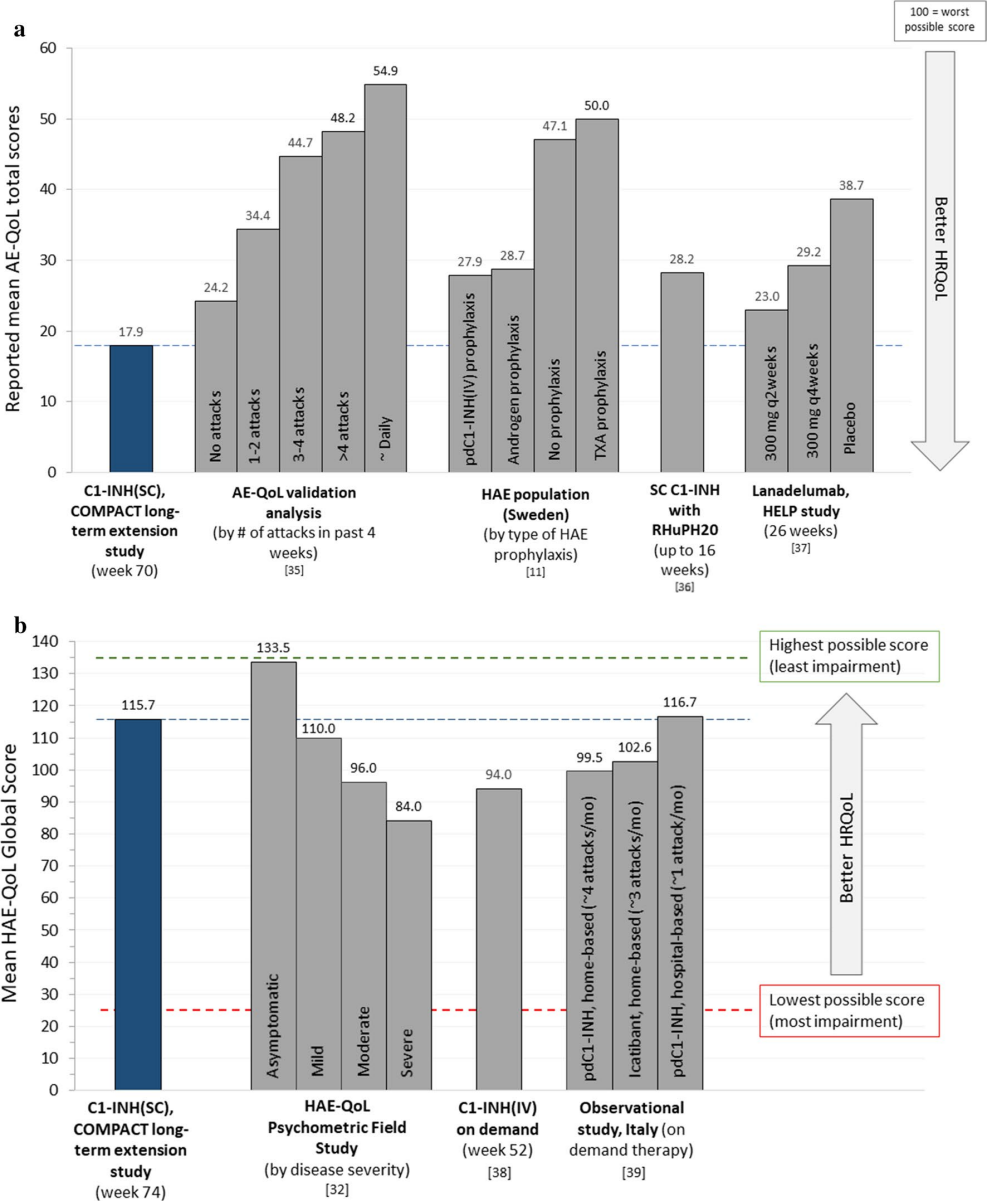
Published mean AE-QoL (**a**) and HAE-QoL (**b**) scores in different patient cohorts. *C1-INH(SC)* subcutaneous C1-inhibitor, *ext* extension, *HAE* hereditary angioedema, *HRQoL* health-related quality of life, *pdC1-INH(IV)* plasma-derived intravenous C1-INH, *TXA* tranexamic acid. *Note* Mean scores for individual domains for the AE-QoL and HAE-QoL are presented in Additional file 6 and Additional file 8.* SC* subcutaneous,* C1-INH* C1-inhibitor,* C1-INH(IV)* intravenous C1-INH,* RhUPH20* recombinant human hyaluronidase,* AE-QoL* Angioedema Quality of Life Questionnaire,* HAE-QoL* hereditary Angioedema Quality of Life Questionnaire

The AE-QoL was administered during the additional 88-week extension period in the US at week 6 (n = 38), week 22 (n = 45), week 46 (n = 43), and week 70 (n = 30). For the total score, the mean at each visit ranged from 13.39 to 17.89 (scale, 0–100, with higher scores indicating greater impairment) (Table 3). Figure 4a illustrates the mean AE-QoL global score at week 70 in comparison with other published AE-QoL data. Scores for functioning and nutrition domains were notably low, ranging from 5.10 to 7.08 for functioning and ranging from 5.92 to 10.76 for nutrition. Mean scores for fears/shame and fatigue/mood ranged from 15.36 to 26.11. Mean scores by individual AE-QoL domain are provided in Additional file 6, which presents mean scores by individual AE-QoL domain along with comparative published data for the same domains in other HAE populations.

**Table 3:** Mean AE-QoL and HAE-QoL scores during the 88-week extension (US only), all C1-INH(SC) doses combined

Domain (range of possible scores)	Extension week 6 (n = 38)	Extension week 22 (n = 45)	Extension week 46 (n = 43)	Extension week 70 (n = 30)
AE-QoL scores^a^, mean (SD) (higher scores = greater impairment)				
Total score (0–100)	13.39 (13.701)	17.68 (17.097)	16.45 (15.761)	17.89 (18.936)
Functioning (0–100)	5.10 (11.881)	7.08 (18.396)	5.96 (15.607)	7.08 (20.351)
Fatigue/mood (0–100)	20.66 (19.734)	26.11 (22.102)	23.14 (20.296)	23.83 (22.116)
Fears/shame (0–100)	15.35 (18.787)	20.09 (22.355)	19.77 (21.499)	22.64 (26.526)
Nutrition (0–100)	5.92 (10.372)	10.56 (18.262)	10.76 (18.416)	10.42 (21.795)

Domain (range of possible scores)	Extension week 26 (n = 40)	Extension week 50 (n = 40)	Extension week 74 (n = 16)
HAE-QoL scores^b^, mean (SD) global score range: 25 to 135 (higher scores = less impairment)			
Global score (25–135)	120.2 (19.15)	122.3 (19.72)	115.7 (26.54)
Physical functioning and health (4–23)	21.0 (3.00)	21.3 (3.05)	20.9 (3.70)
Disease related stigma (3–15)	13.8 (2.24)	13.7 (2.37)	13.6 (2.66)
Emotional role and social functioning (4–20)	18.4 (2.81)	18.5 (2.86)	18.3 (3.18)
Concern about offspring (2–10)	7.9 (2.91)	8.3 (2.66)	7.8 (2.65)
Perceived control over Illness (4–20)	16.7 (4.23)	17.4 (4.12)	15.5 (6.03)
Mental health (4–24)	21.2 (3.92)	21.9 (3.49)	20.3 (4.84)
Treatment difficulties (4–23)	21.4 (2.87)	21.3 (3.26)	19.4 (5.40)

These assessments were not performed at baseline

*AE-QoL* Angioedema Quality of Life Questionnaire,* HAE* hereditary angieodema,* HAE-QoL* hereditary angeiodema quality of life questionnaire,* HRQoL* health-related quality of life,* C1-INH(SC)* subcutaneous C1-inhibitor,* US* United States,* SD* standard deviation

^a^The AE-QoL [31, 35] is a validated angioedema-specific instrument comprised of 17 questions based on a recall period of 4 weeks. For the total and each domain, scores can range from 0 to 100 (higher scores indicate greater impairment)

^b^The HAE-QoL [32] is a HRQoL questionnaire specifically designed for studying the impact of HAE due to C1-INH(SC) deficiency on adult patients’ quality of life. It consists of 25 items assigned to 7 dimensions (3 or 4 items per dimension); each item scored from 1 to 5 or 1 to 6. The global score reflects the sum of all 25 individual item scores (higher scores indicate better outcomes)

Median AE-QoL scores by visit are provided in Additional file 7. For total score, the median values ranged from 10.29 to 14.71.

### HAE-QoL

The HAE-QoL was administered during the additional 88-week extension period in the US at week 26 (n = 40), week 50 (n = 40), and week 74 (n = 16). Mean global scores at these visits, which can range from 25 to 135 (higher scores indicating better health status), ranged from 115.7 to 122.3 (Table 3). Figure 4b illustrates the mean HAE-QoL global score at week 74 in comparison with other published HAE-QoL data. For individual HAE-QoL domains, mean values reflected good HRQoL and remained generally stable at all assessment time points (Table 3).

Median HAE-QoL scores by visit are provided in Additional file 7. For global score, the median values ranged from to 126.0 to 128.5. Mean scores by individual HAE-QoL domain are provided in Additional file 8, which also presents comparative published data for the same domains in other HAE populations.

## Discussion

We have previously reported clinically relevant improvements in HRQoL experienced by patients using self-administered prophylaxis with C1-INH(SC) over a period of 4 months in the randomized, double-blind, placebo-controlled, crossover design COMPACT phase III study [25]. This analysis of data from the COMPACT open-label extension study now encompasses a substantially longer observation period, more than two years in some patients, and provides data to evaluate the impact of long-term treatment with C1-INH(SC) on various HRQoL and productivity measures. This study incorporated a battery of four generic, commonly used HRQoL assessment tools (EQ-5D, HADS, WPAI, TSQM), as well as two recently developed disease-specific instruments (AE-QoL, HAE-QoL). In both the double-blind COMPACT study and the open-label extension study, patient eligibility required a minimum attack frequency of two or more attacks per month prior to study entry.

Considering that two-thirds (63%) of patients in the open-label study were on a prophylactic treatment prior to study participation, the significant improvements from baseline at almost every measured HRQoL outcome (in the twice weekly 60 IU/kg C1-INH[SC] arm) are strong evidence for the benefits of C1-INH(SC) prophylaxis as well as the importance of reducing the burden of attacks. The significant improvements included important items in the EQ-5D, HADS, WPAI, and TSQM instruments such as overall quality of life, depression, anxiety, and work productivity domains. The only exception was absenteeism, which was already very low at baseline in the study population. Further, beyond achieving significance based on statistical considerations (CI values), many of the HRQoL outcome improvements met criteria for a clinically important difference in a substantial percentage of patients, which was especially notable given that mean baseline values were already higher (better) than US population norms for the EQ-5D (Health State Value and VAS) and within the scoring range interpreted as “normal” for the HADS depression and anxiety scales.

The EQ-5D is a well-recognized, generic HRQoL instrument frequently used in HAE research [5, 10, 17] and is commonly used for cost effectiveness assessments. The EQ-5D assesses five dimensions, including mobility, self-care, usual activities, pain/discomfort, and anxiety/depression. One of two scoring metrics for the EQ-5D is the Health State Value, for which scores range from 0 (dead) to 1 (full health). The baseline mean Health State Value score in the current study population was already very good (0.90) and higher than reported as a US population norm (0.825) [34]. Yet, at the completion of 52 or 140 weeks of open-label C1-INH(SC) prophylaxis, the mean EQ-5D Health State Value score was even higher (0.95). This increase of 0.05 is within the range considered to reflect a minimally important difference for this tool (0.037–0.069) [40]. Further, it was an important finding that with multiple assessments over periods of one or two years and even longer in many patients, mean EQ-5D scores remained high and consistent from visit to visit, most notably starting at week 9. To our knowledge, this is the first study to report EQ-5D Health State Value improvements with HAE prophylaxis. In one other study involving analysis of EQ-5D responses from patients in the Swedish HAE registry during 2016, patients were grouped according to whether they were using any type of HAE prophylaxis or not [10]. No significant difference was noted for mean EQ-5D Health State Value scores between 26 patients who were using prophylaxis (androgens [n = 18], tranexamic acid [n = 4], or plasma-derived C1-INH(IV) [n = 4]) as compared with 23 patients not using prophylaxis (mean scores, 0.88 vs 0.78, respectively).

The other scoring metric for the EQ-5D is the VAS, which ranges from 0 (worst) to 100 (best imaginable health state). In the current study, the mean EQ-5D VAS score increased significantly from baseline to end of study in the C1-INH 60 IU/kg group (79.75 to 87.20, difference = 7.45). In the 26-week HELP study, which assessed the prophylactic efficacy of the monoclonal antibody lanadelumab, end-of-study mean EQ-5D VAS scores were 83.15 (300 mg Q 2 weeks) and 82.46 (300 mg Q 4 weeks), representing changes from baseline of + 2.08 and − 0.33, respectively, neither of which was statistically significant. The findings of these two studies should be compared with caution, given unknown effects of differences between the two studies with regard to study design, study duration, patient populations, and potentially other factors on the outcomes. Further research will be required to assess potential HRQoL differences between treatments.

The newer symptom-specific (AE-QoL) and disease-specific (HAE-QoL) HRQoL instruments were evaluated only during the extra 88-week extension phase. Patients had been using open-label C1-INH(SC) for at least 52 weeks prior to being evaluated with these instruments so there were no “baseline” measurements. Assessment of the AE-QoL and HAE-QoL findings showed stable mean values for these scores over periods of many weeks. Figure 4a compares the mean AE-QoL score from C1-INH(SC) users at the last assessment of the current study with published data from other studies utilizing the AE-QoL in patients with C1-INH-HAE. The mean score reported for patients using C1-INH(SC) (17.9) was lower (better) than all other published values to date, including those reported in a Swedish population with C1-INH-HAE using other types of prophylaxis [10]. Further, in the current study, mean AE-QoL total scores changed little over time and at all time points were below (better than) the reported mean score for angioedema patients experiencing no attacks in the past 4 weeks in the AE-QoL validation study (24.2) [35]. The mean AE-QoL total score in C1-INH(SC) users in the current study (17.9) was also lower than previously reported in the HELP study with lanadelumab given every 2 weeks (23.0) or every 4 weeks (29.2) [37]. It was also lower than reported with subcutaneous administration of another C1-INH preparation (C1-INH combined with rHuPH20) (28.2) [36]. Thus, the AE-QoL scores in the current study within the context of other published data were highly indicative of good HRQoL for patients with C1-INH-HAE. In the current study, mean global HAE-QoL scores at each visit were high, close to the maximum (best) possible score of 135, and changed little over months of therapy. The mean HAE-QoL global score at week 74 was higher (better) than scores in the psychometric field study of the HAE-QoL for patients with any severity of symptomatic HAE (Fig. 4b). We were unable to locate any other published studies using the HAE-QoL specifically in patients managed with prophylactic HAE medications. A small single-center study in Italy [38] utilized the HAE-QoL to evaluate HRQoL in 15 patients using C1-INH(IV) as on demand treatment of HAE attacks. In that study, the patients experienced a median of 4 attacks/patient over a 1-year study period. The mean global HAE-QoL score increased (improved) from 88.6 at baseline to 94.0 after 12 months of on demand treatment with C1-INH(IV), a value that was still markedly lower than the mean global HAE-QoL scores achieved in the current study with C1-INH(SC) prophylaxis (mean values per visit, range of 115.7 to 122.3). An observational study in Italy reported HAE-QoL scores for three groups of patients with C1-INH-HAE grouped based on the type of therapy they were using to treat attacks: hospital-based treatment with C1-INH(IV); home-based treatment with SC icatibant; or home-based treatment with C1-INH(IV) [39]. There was no significant difference in mean HAE-QoL scores between the groups, although there was a trend toward better HRQoL in the hospital-based therapy patients (Fig. 4b). The hospital-based therapy group also had a significantly lower attack rate (~ 1 per month) compared to the other two groups (~ 3–4 attacks per month). It should be noted that one-fifth of the patients (12 of 56; 21.4%) were younger than 15 years old, an age group for which the HAE-QoL has not been validated. Regardless, these data seem to reinforce the importance of attack frequency on HRQoL in patients with C1-INH-HAE. Again, all comparisons of quality of life measures between studies should be made with caution given numerous potential differences and variations between populations and study design.

### Limitations

The findings of our study should be interpreted with certain limitations in mind. This was a pre-specified analysis of exploratory, subject-reported outcomes from an open-label clinical trial. There were no specific efforts undertaken to follow-up on missing data and analysis was done on as reported data only. Therefore, findings of these analyses are to be interpreted as observational, since the study had no scientific hypothesis on an assumed effect size of C1-INH(SC) on quality of life measures. Also the MCID analysis was a post hoc analysis and MCIDs have not been established in patients with HAE for the patient-reported outcomes evaluated in this study. Nevertheless the 0.5 SD metric for MCID can approximate the MCID in many situations.

The HRQoL measures were captured at a limited number of time points throughout this long study, often with weeks to months in between assessments. Not all patients completed all questionnaires at all scheduled time points and scores were not adjusted for the smaller numbers of responses in such situations. Ten subjects were younger than 17 years old; all of them contributed responses for the EQ-5D, HADS, and TSQM, and half of them contributed responses for the AE-QoL and HAE-QoL. While the HADS instrument has been validated in children between 12–17 years of age [41], the EQ-5D, HAE-QoL, and AE-QoL have not been validated for adolescents and younger patients with HAE. However, study site personnel made every effort to ensure that study subjects younger than 18 years old and their caregivers understood all questions related to HAE and its impact on quality of life. The COMPACT trial was an international project involving patients from 11 different countries, and it is possible that HRQoL scores could have been influenced by geographical location; however, it should be noted that the questionnaires that were used have been validated in each of the countries represented and country-specific effects would not be expected. The AE-QoL and HAE-QoL assessments were administered only to patients in the US. This was an open-label trial with no control group, thus the influence of non-treatment- related factors leading to changes in HRQoL cannot be ruled out. Further, “end of study” change from baseline assessments reflected two different time points (52 weeks or 140 weeks), depending on whether or not a subject participated in the 88-week extension phase. Finally, COMPACT trial entry criteria required patients to have a minimum baseline HAE attack frequency and that they be considered appropriate candidates for self-injection; thus, the study population may not be fully representative of all individuals with C1-INH-HAE.

## Conclusion

In patients with C1-INH-HAE and frequent angioedema attacks managed long-term with open-label C1-INH(SC) 60 IU/kg twice weekly, a battery of HRQoL assessments revealed clinically meaningful and sustained improvements from baseline in overall quality of life, anxiety, depression, productivity, and satisfaction with therapy. It is clinically intuitive to assume that these improvements were largely influenced by the clinical efficacy outcomes reported with this regimen during the open-label extension study, namely a median annualized attack rate of 1.0 [26] and median rescue medication usage of 0.0. These findings reinforce the opportunity for improving clinically important HRQoL parameters in parallel with effective attack rate reduction when patients are transitioned to effective prophylactic therapies such as C1-INH(SC).

## Supplementary Information

**Additional file 1**. Mean/median changes in patient-reported HRQoL outcomes from baseline to end of study in patients treated with C1-INH(SC) 40 or 60 IU/kg twice weekly.

**Additional file 2**. Mean (SD) EQ-5D scores by study visit, all C1-INH(SC) combined.

**Additional file 3**. Mean (SD) HADS scores by study visit, all C1-INH(SC) combined.

**Additional file 4**. Mean (SD) WPAI scores by study visit, all C1-INH(SC) combined.

**Additional file 5**. Mean (SD) TSQM scores by study visit, all C1-INH(SC) combined.

**Additional file 6**. Mean AE-QoL scores by individual domain along with comparative published data for the same domains in other HAE populations.

**Additional file 7**. Median AE-QoL and HAE-QoL score at different time points during the 88-week extension period, all C1-INH(SC) doses combined (US only).

**Additional file 8**. Mean HAE-QoL scores by individual domain along with comparative published data for the same domains in other HAE populations.

## Data Availability

The datasets used and/or analyzed during the current study are available from the corresponding author on reasonable request.

## Abbreviations

AEs: Adverse events
AE-QoL: Angioedema Quality of Life Questionnaire
C1-INH: C1-inhibitor
C1-INH-HAE: C1-INH deficiency
CI: Confidence interval
EQ-5D: European quality of life-5 dimensions questionnaire
EQ-5D-3L: European quality of life-5 dimensions questionnaire 3-level version
HRQoL: Health-related quality of life
HAE: Hereditary angioedema
HAE-QoL: Hereditary Angioedema Quality of Life Questionnaire
HADS: Hospital anxiety and depression scale
MCID: Minimal clinically important differences
C1-INH(SC): Subcutaneous C1-INH
TSQM: Treatment satisfaction questionnaire for medication
VAS: Visual analog scale
WPAI: Work productivity and activity impairment questionnaire
C1-INH(IV): Intravenous C1-INH
RhUPH20: Recombinant human hyaluronidase
TP: Treatment period
US: United States
BMI: Body mass index
SD: Standard deviation

## References

[1] Cicardi M, Aberer W, Banerji A, Bas M, Bernstein JA, Bork K (2014). Classification, diagnosis, and approach to treatment for angioedema: consensus report from the Hereditary Angioedema International Working Group. Allergy.

[2] Lumry WR, Castaldo AJ, Vernon MK, Blaustein MB, Wilson DA, Horn PT (2010). The humanistic burden of hereditary angioedema: impact on health-related quality of life, productivity, and depression. Allergy Asthma Proc.

[3] Wilson DA, Bork K, Shea EP, Rentz AM, Blaustein MB, Pullman WE (2010). Economic costs associated with acute attacks and long-term management of hereditary angioedema. Ann Allergy Asthma Immunol.

[4] Aygören-Pürsün E, Bygum A, Beusterien K, Hautamaki E, Sisic Z, Wait S (2014). Socioeconomic burden of hereditary angioedema: results from the hereditary angioedema burden of illness study in Europe. Orphanet J Rare Dis.

[5] Nordenfelt P, Dawson S, Wahlgren CF, Lindfors A, Mallbris L, Björkander J (2014). Quantifying the burden of disease and perceived health state in patients with hereditary angioedema in Sweden. Allergy Asthma Proc.

[6] Engel-Yeger B, Farkas H, Kivity S, Veszeli N, Kőhalmi KV, Kessel A (2017). Health-related quality of life among children with hereditary angioedema. Pediatr Allergy Immunol.

[7] Huang SW (2004). Results of an on-line survey of patients with hereditary angioedema. Allergy Asthma Proc.

[8] Bygum A (2014). Hereditary angioedema—consequences of a new treatment paradigm in Denmark. Acta Derm Venereol.

[9] Fouche A, Saunders EF, Craig T (2014). Depression and anxiety in patients with hereditary angioedema. Ann Allergy Asthma Immunol.

[10] Nordenfelt P, Nilsson M, Lindfors A, Wahlgren CF, Björkander J (2017). Health-related quality of life in relation to disease activity in adults with hereditary angioedema in Sweden. Allergy Asthma Proc.

[11] Bouillet L, Launay D, Fain O, Boccon-Gibod I, Laurent J, Martin L (2013). Hereditary angioedema with C1 inhibitor deficiency: clinical presentation and quality of life of 193 French patients. Ann Allergy Asthma Immunol.

[12] Bygum A, Andersen KE, Mikkelsen CS (2009). Self-administration of intravenous C1-inhibitor therapy for hereditary angioedema and associated quality of life benefits. Eur J Dermatol.

[13] Bewtra AK, Levy RJ, Jacobson KW, Wasserman RL, Machnig T, Craig TJ (2012). C1-inhibitor therapy for hereditary angioedema attacks: prospective patient assessments of health-related quality of life. Allergy Asthma Proc.

[14] Gomide MA, Toledo E, Valle SO, Campos RA, França AT, Gomez NP (2013). Hereditary angioedema: quality of life in Brazilian patients. Clinics (Sao Paulo).

[15] Bygum A, Aygören-Pürsün E, Beusterien K, Hautamaki E, Sisic Z, Wait S (2015). Burden of illness in hereditary angioedema: a conceptual model. Acta Derm Venereol.

[16] Banerji A (2013). The burden of illness in patients with hereditary angioedema. Ann Allergy Asthma Immunol.

[17] Aygören-Pürsün E, Bygum A, Beusterien K, Hautamaki E, Sisic Z, Boysen HB (2016). Estimation of EuroQol 5-dimensions health status utility values in hereditary angioedema. Patient Prefer Adherence.

[18] Cardarelli W (2013). Managed care implications of hereditary angioedema. Am J Manag Care.

[19] Longhurst H, Bygum A (2016). The humanistic, societal, and pharmaco-economic burden of angioedema. Clin Rev Allergy Immunol.

[20] Jindal NL, Harniman E, Prior N, Perez-Fernandez E, Caballero T, Betschel S (2017). Hereditary angioedema: health-related quality of life in Canadian patients as measured by the SF-36. Allergy Asthma Clin Immunol.

[21] Maurer M, Magerl M, Ansotegui I, Aygören-Pürsün E, Betschel S, Bork K (2018). The international WAO/EAACI guideline for the management of hereditary angioedema—the 2017 revision and update. Allergy.

[22] Weller K, Krüger R, Maurer M, Magerl M (2016). Subcutaneous self-injections of C1 inhibitor: an effective and safe treatment in a patient with hereditary angio-oedema. Clin Exp Dermatol.

[23] Zuraw BL, Cicardi M, Longhurst HJ, Bernstein JA, Li HH, Magerl M (2015). Phase II study results of a replacement therapy for hereditary angioedema with subcutaneous C1-inhibitor concentrate. Allergy.

[24] Longhurst H, Cicardi M, Craig T, Bork K, Grattan C, Baker J (2017). Prevention of hereditary angioedema attacks with a subcutaneous C1 Inhibitor. N Engl J Med.

[25] Lumry WR, Craig T, Zuraw B, Longhurst H, Baker J, Li HH (2018). Health-related quality of life with subcutaneous C1-inhibitor for prevention of attacks of hereditary angioedema. J Allergy Clin Immunol Pract.

[26] Craig T, Zuraw B, Longhurst H, Cicardi M, Bork K, Grattan C (2019). Long-term outcomes with subcutaneous C1-inhibitor replacement therapy for prevention of hereditary angioedema attacks. J Allergy Clin Immunol Pract.

[27] EuroQol Research Foundation (2015). EQ-5D-3L user guide, version 5.1.

[28] Zigmond AS, Snaith RP (1983). The hospital anxiety and depression scale. Acta Psychiatr Scand.

[29] Reilly MC, Zbrozek AS, Dukes EM (1993). The validity and reproducibility of a work productivity and activity impairment instrument. Pharmacoeconomics.

[30] Atkinson MJ, Sinha A, Hass SL, Colman SS, Kumar RN, Brod M (2004). Validation of a general measure of treatment satisfaction, the Treatment Satisfaction Questionnaire for Medication (TSQM), using a national panel study of chronic disease. Health Qual Life Outcomes.

[31] Weller K, Groffik A, Magerl M, Tohme N, Martus P, Krause K (2012). Development and construct validation of the angioedema quality of life questionnaire. Allergy.

[32] Prior N, Remor E, Pérez-Fernández E, Caminoa M, Gómez-Traseira C, Gayá F (2016). Psychometric field study of hereditary angioedema quality of life questionnaire for adults: HAE-QoL. J Allergy Clin Immunol Pract.

[33] Norman GR, Sloan JA, Wyrwich KW (2003). Interpretation of changes in health-related quality of life: the remarkable universality of half a standard deviation. Med Care.

[34] Janssen B, Szende A, Szende A, Janssen B, Cabases J (2014). Population norms for the EQ-5D. Self-reported population health: an international perspective based on EQ-5D.

[35] Weller K, Magerl M, Peveling-Oberhag A, Martus P, Staubach P, Maurer M (2016). The Angioedema Quality of Life Questionnaire (AE-QoL) - Assessment of sensitivity to change and minimal clinically important difference. Allergy..

[36] Weller K, Maurer M, Fridman M, Supina D, Schranz J, Magerl M (2017). Health-related quality of life with hereditary angioedema following prophylaxis with subcutaneous C1-inhibitor with recombinant hyaluronidase. Allergy Asthma Proc..

[37] Lanadelumab (Takhzyro ®) German Dossier. Accessed from: https://www.g-ba.de/downloads/92-975-2914/2019-02-01_Modul4A_Lanadelumab.pdf.

[38] Zanichelli A, Azin GM, Cristina F, Vacchini R, Caballero T (2018). Safety, effectiveness, and impact on quality of life of self-administration with plasma-derived nanofiltered C1 inhibitor (Berinert®) in patients with hereditary angioedema: the SABHA study. Orphanet J Rare Dis..

[39] Squeglia V, Barbarino A, Bova M, Gravante C, Petraroli A, Spadaro G (2016). High attack frequency in patients with angioedema due to C1-inhibitor deficiency is a major determinant in switching to home therapy: a real-life observational study. Orphanet J Rare Dis..

[40] McClure NS, Sayah FA, Xie F, Luo N, Johnson JA (2017). Instrument-defined estimates of the minimally important difference for EQ-5D-5L index scores. Value Health..

[41] White D, Leach C, Sims R, Atkinson M, Cottrell D (1999). Validation of the Hospital Anxiety and Depression Scale for use with adolescents. Br J Psych..

