# Correction to: Long-term health-related quality of life in patients treated with subcutaneous C1-inhibitor replacement therapy for the prevention of hereditary angioedema attacks: findings from the COMPACT open-label extension study

**DOI:** 10.1186/s13023-021-01975-2

**Published:** 2021-07-28

**Authors:** William R. Lumry, Bruce Zuraw, Marco Cicardi, Timothy Craig, John Anderson, Aleena Banerji, Jonathan A. Bernstein, Teresa Caballero, Henriette Farkas, Richard G. Gower, Paul K. Keith, Donald S. Levy, H. Henry Li, Markus Magerl, Michael Manning, Marc A. Riedl, John-Philip Lawo, Subhransu Prusty, Thomas Machnig, Hilary Longhurst, the COMPACT Investigators

**Affiliations:** 1 AARA Research Center, 10100 N. Central Expressway, Suite 100, Dallas, TX 75231 USA; 2 Department of Medicine, UC San Diego, La Jolla, CA USA; 3 University of Milan, Milan, Italy; 4 Penn State University College of Medicine, Hershey, PA USA; 5 Clinical Research Center of Alabama, Birmingham, AL USA; 6 Massachusetts General Hospital, Boston, MA USA; 7 University of Cincinnati College of Medicine and Bernstein Clinical Research Center, LLC, Cincinnati, OH USA; 8 Allergy Department, Hospital La Paz Institute for Health Research (IdiPaz), Biomedical Research Network On Rare Diseases (CIBERER U754), Madrid, Spain; 9 Hungarian Angioedema Reference Center, 3Rd Department of Internal Medicine, Semmelweis University, Budapest, Hungary; 10 Maryclif Clinical Research, Spokane, WA USA; 11 McMaster University, Hamilton, ON USA; 12 UC Irvine, Orange, CA USA; 13 Institute for Asthma and Allergy, Chevy Chase, MD USA; 14 Department of Dermatology and Allergy, Charité, Universitätsmedizin Berlin, Berlin, Germany; 15 Medical Research of Arizona, Scottsdale, AZ USA; 16 Division of Rheumatology, Allergy and Immunology, University of California, San Diego, La Jolla, CA USA; 17 CSL Behring GmbH, Emil-von-Behring-Strasse 76, Marburg, Germany; 18 University College Hospital, London, UK; 19 Auckland District Health Board, Auckland, New Zealand

## Correction to: Orphanet J Rare Dis (2021) 16:86 10.1186/s13023-020-01658-4

Following the publication of the original article [1] the authors informed us of an error in the second paragraph under ‘Study population’ of the ‘Results’ section.

The paragraph should read:

“Half of the patients in the study (n = 64, 50.8%) were participants in the double-blind COMPACT study, thus were using C1-INH(SC) prophylaxis prior to continuing into the open-label study (Table 1). An additional 15 patients (11.9%) were using other forms of HAE prophylaxis (intravenous C1-INH [C1-INH[IV)] or oral danazol) within the 3-month period prior to the open-label study”.

The original article has already been updated as above.
