# Supplementary material for: Long-term health-related quality of life in patients treated with subcutaneous C1-inhibitor replacement therapy for the prevention of hereditary angioedema attacks: findings from the COMPACT open-label extension study
Source: Orphanet J Rare Dis. 2021 Feb 15;16:86. doi: 10.1186/s13023-020-01658-4 (PMC7885603; doi:10.1186/s13023-020-01658-4)
Supplement: Supplementary file 1 — Additional file 1. Mean/median changes in patient-reported HRQoL outcomes from baseline to end of study in patients treated with C1-INH(SC) 40 or 60 IU/kg twice weekly. [file 13023_2020_1658_MOESM1_ESM.docx]

**Additional file 1.** Mean/median changes in patient-reported HRQoL outcomes from baseline to end of study in patients treated with C1-INH(SC) 40 or 60 IU/kg twice weekly

|  | C1-INH(SC) 40 IU/kg  vs baseline | | | C1-INH(SC) 60 IU/kg  vs baseline | | |
| --- | --- | --- | --- | --- | --- | --- |
|  | n | Diff | 95% CI | n | Diff | 95% CI |
| **Mean** | | | | | | |
| EQ-5D |  |  |  |  |  |  |
| Health State Value | 48 | 0.03 | (-0.00, 0.06) | 44 | 0.07 | **(0.01, 0.12)** |
| VAS | 48 | 4.33 | **(-0.13, 8.80)** | 44 | 7.45 | **(3.29, 11.62)** |
|  |  |  |  |  |  |  |
| HADS |  |  |  |  |  |  |
| Depression | 48 | -0.67 | (-1.57, 0.24) | 44 | -0.95 | **(-1.57, -0.34)** |
| Anxiety | 48 | -1.23 | **(-2.21, -0.25)** | 44 | -1.23 | **(-2.08, -0.38)** |
|  |  |  |  |  |  |  |
| TSQM |  |  |  |  |  |  |
| Effectiveness | 44 | 10.98 | **(3.96, 18.01)** | 38 | 19.74 | **(7.94, 31.54)** |
| Convenience | 44 | 11.11 | **(6.47, 15.75)** | 38 | 6.14 | (-0.41, 12.69) |
| Overall Satisfaction | 44 | 10.80 | **(4.53, 17.06)** | 38 | 18.93 | **(10.68, 27.18)** |
|  |  |  |  |  |  |  |
| WPAI |  |  |  |  |  |  |
| Absenteeism | 28 | -2.08 | (-12.03, 7.87) | 24 | -8.17 | (-16.72, 0.37) |
| Presenteeism | 27 | -6.30 | (-19.65, 7.06) | 24 | -23.33 | **(-34.86, -11.81)** |
| Work Productivity Loss | 27 | -6.46 | (-20.60, 7.69) | 24 | -26.68 | **(-39.92, -13.44)** |
| Activity Impairment | 48 | -12.71 | **(-21.63, -3.79)** | 44 | -16.14 | **(-26.36, -5.91)** |
|  |  |  |  |  |  |  |
| **Median** | | | | | | |
| EQ-5D |  |  |  |  |  |  |
| Health State Value | 48 | 0.00 | (0.00, 0.00) | 44 | 0.00 | (0.00, 0.00) |
| VAS | 48 | 2.50 | (0.00, 8.00) | 44 | 2.50 | (0.00, 10.00) |
|  |  |  |  |  |  |  |
| HADS |  |  |  |  |  |  |
| Depression | 48 | 0.00 | (-1.00, 0.00) | 44 | -1.00 | (-1.00, 0.00) |
| Anxiety | 48 | -1.00 | (-2.00, 0.00) | 44 | -1.00 | (-2.00, 0.00) |
|  |  |  |  |  |  |  |
| TSQM |  |  |  |  |  |  |
| Effectiveness | 44 | 5.56 | (0.00, 16.67) | 38 | 16.67 | **(11.11, 33.33)** |
| Convenience | 44 | 8.33 | **(5.56, 16.67)** | 38 | 5.56 | (0.00, 11.11) |
| Overall Satisfaction | 44 | 5.56 | (0.00, 16.67) | 38 | 9.72 | **(5.56, 22.22)** |
|  |  |  |  |  |  |  |
| WPAI |  |  |  |  |  |  |
| Absenteeism | 28 | 0.00 | (0.00, 0.00) | 24 | 0.00 | (0.00, 0.00) |
| Presenteeism | 27 | 0.00 | (-10.00, 0.00) | 24 | -10.00 | (-30.00, 0.00) |
| Work Productivity Loss | 27 | 0.00 | (-10.00, 0.00) | 24 | -10.69 | (-28.95, 0.00) |
| Activity Impairment | 48 | 0.00 | (-10.00, 0.00) | 44 | -10.00 | (-30.00, 0.00) |

Bold values indicate statistical significance as determined by a CI that does not include zero.

C1-INH(SC), subcutaneous C1-inhibitor; CI, confidence interval; EQ-5D, European Quality of Life-5 Dimensions; HADS, Hospital Anxiety and Depression Scale; TSQM, Treatment Satisfaction Questionnaire for Medication; WPAI, Work Productivity and Activity Impairment Questionnaire.
