# Supplementary material for: Long-term health-related quality of life in patients treated with subcutaneous C1-inhibitor replacement therapy for the prevention of hereditary angioedema attacks: findings from the COMPACT open-label extension study
Source: Orphanet J Rare Dis. 2021 Feb 15;16:86. doi: 10.1186/s13023-020-01658-4 (PMC7885603; doi:10.1186/s13023-020-01658-4)
Supplement: Supplementary file 4 — Additional file 4. Mean (SD) WPAI scores by study visit, all C1-INH(SC) combined. [file 13023_2020_1658_MOESM4_ESM.docx]

**Additional file 4.** Mean (SD) WPAI scores by study visit, all C1-INH(SC) combined

A)


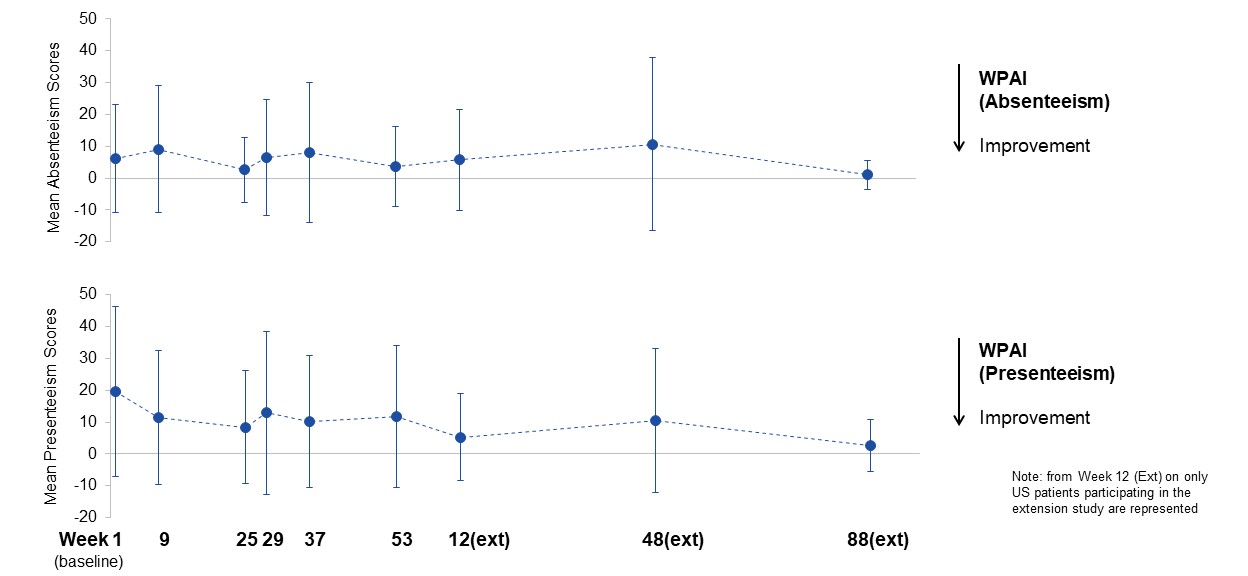


| Number of patients completing assessments | **Week** | | | | | | | | |
| --- | --- | --- | --- | --- | --- | --- | --- | --- | --- |
|  | **1** | **9** | **25** | **29** | **37** | **53** | **12(ext)** | **48(ext)** | **88(ext)** |
| Absenteeism, n | 69 | 66 | 66 | 68 | 66 | 64 | 27 | 28 | 30 |
| Presenteeism, n | 68 | 66 | 66 | 68 | 64 | 64 | 27 | 27 | 30 |

**B)**
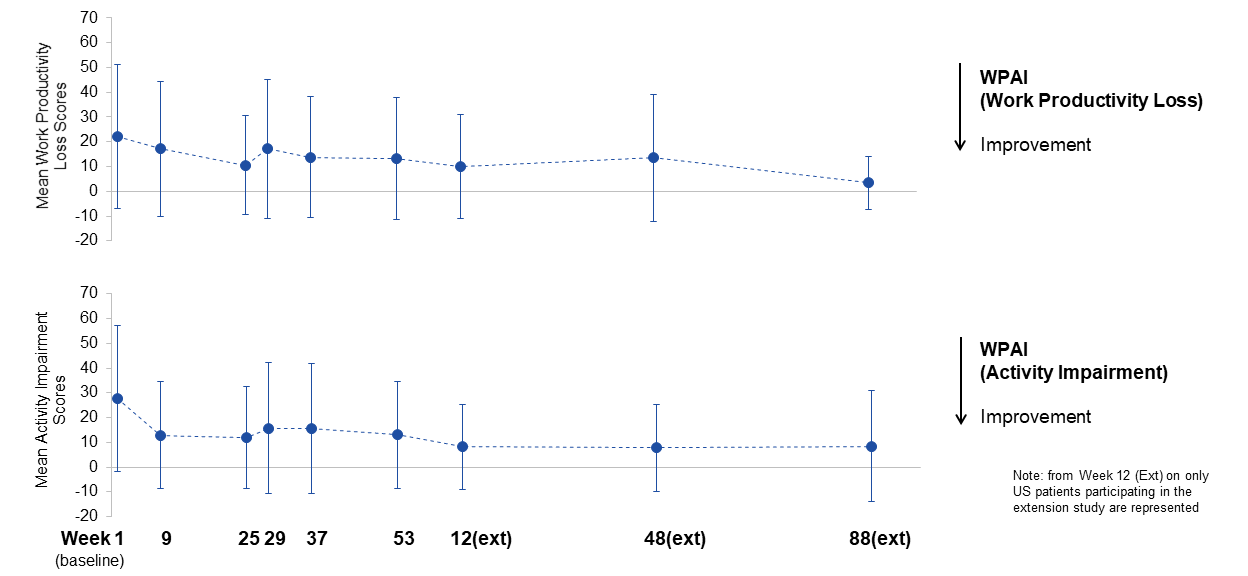


| Number of patients completing assessments | **Week** | | | | | | | | |
| --- | --- | --- | --- | --- | --- | --- | --- | --- | --- |
|  | **1** | **9** | **25** | **29** | **37** | **53** | **12(ext)** | **48(ext)** | **88(ext)** |
| Productivity Loss, n | 68 | 66 | 66 | 68 | 64 | 64 | 27 | 27 | 30 |
| Activity Impairment, n | 114 | 112 | 107 | 105 | 107 | 92 | 44 | 38 | 39 |

C1-INH(SC), subcutaneous C1-inhibitor; WPAI, Work Productivity and Activity Impairment Questionnaire
