# Supplementary material for: Long-term health-related quality of life in patients treated with subcutaneous C1-inhibitor replacement therapy for the prevention of hereditary angioedema attacks: findings from the COMPACT open-label extension study
Source: Orphanet J Rare Dis. 2021 Feb 15;16:86. doi: 10.1186/s13023-020-01658-4 (PMC7885603; doi:10.1186/s13023-020-01658-4)
Supplement: Supplementary file 6 — Additional file 6. Mean AE-QoL scores by individual domain along with comparative published data for the same domains in other HAE populations. [file 13023_2020_1658_MOESM6_ESM.docx]

**Additional file 6.** Mean AE-QoL scores by individual domain along with comparative published data for the same domains in other HAE populations

A) Functioning


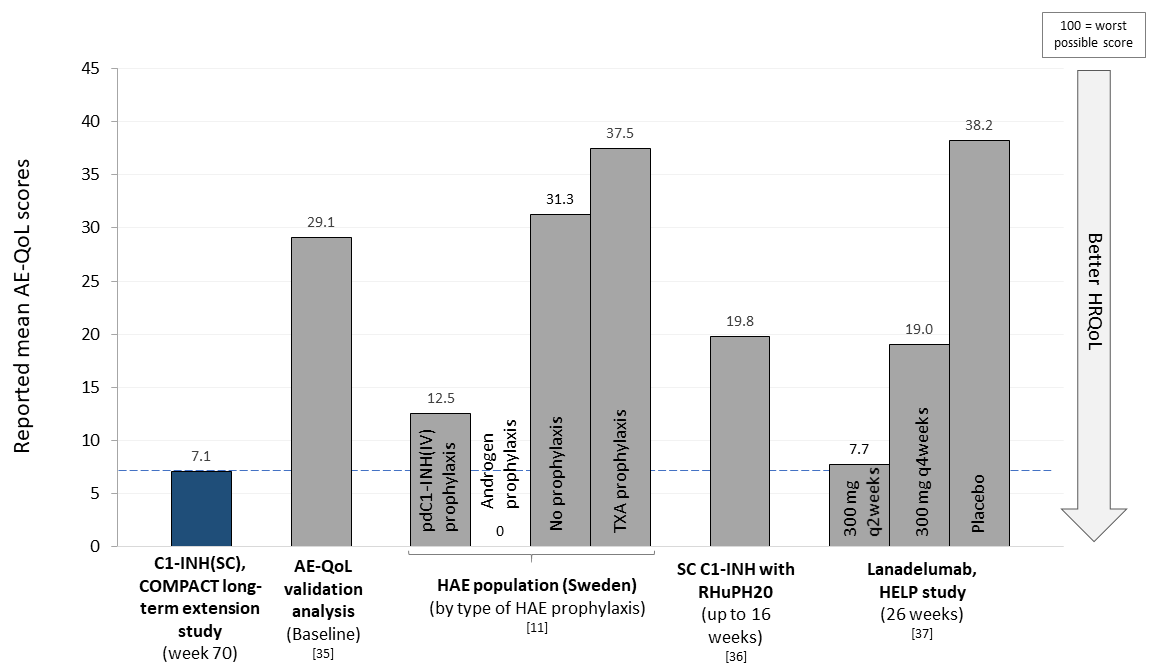


B) Fatigue/Mood


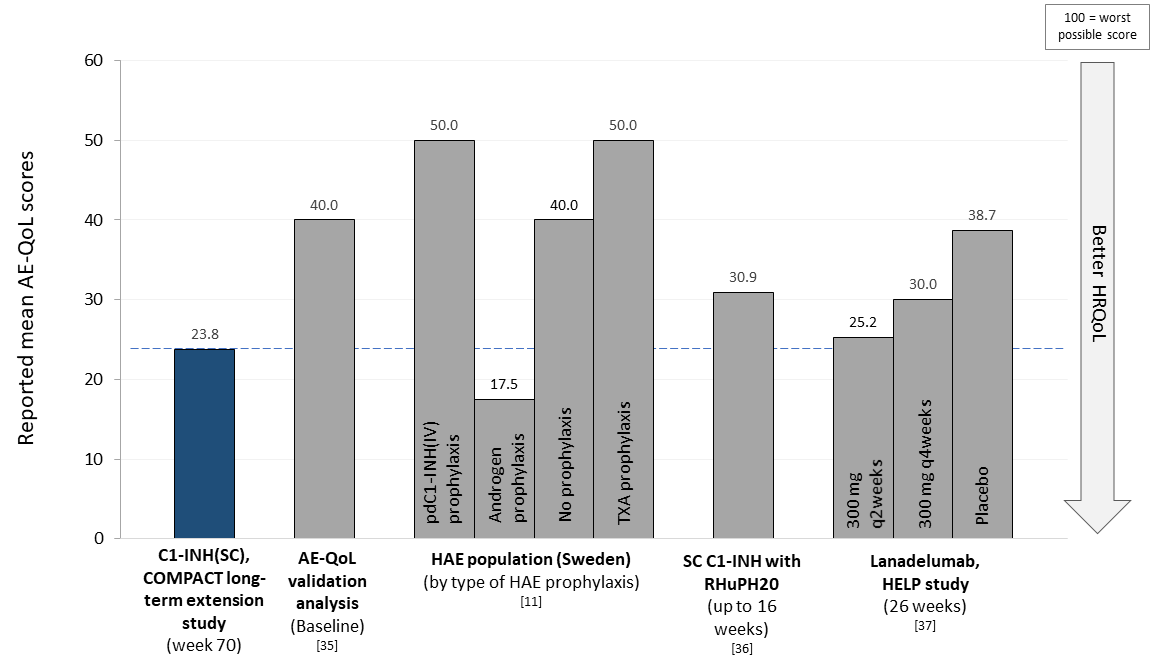


C) Fears/Shame


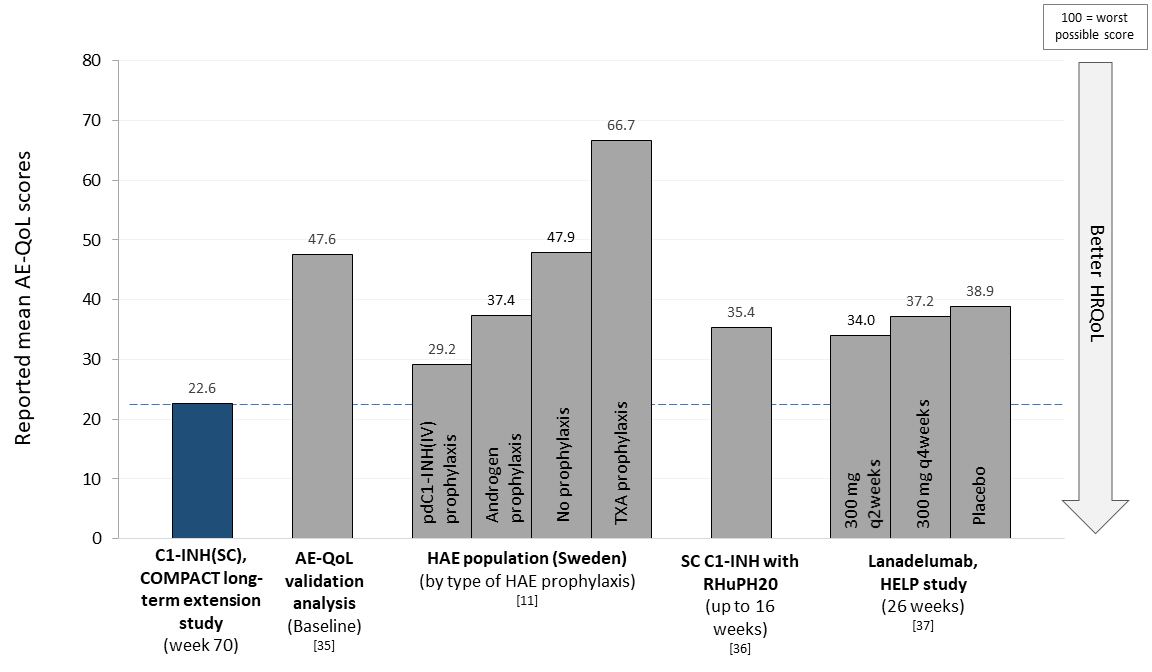


D) Food


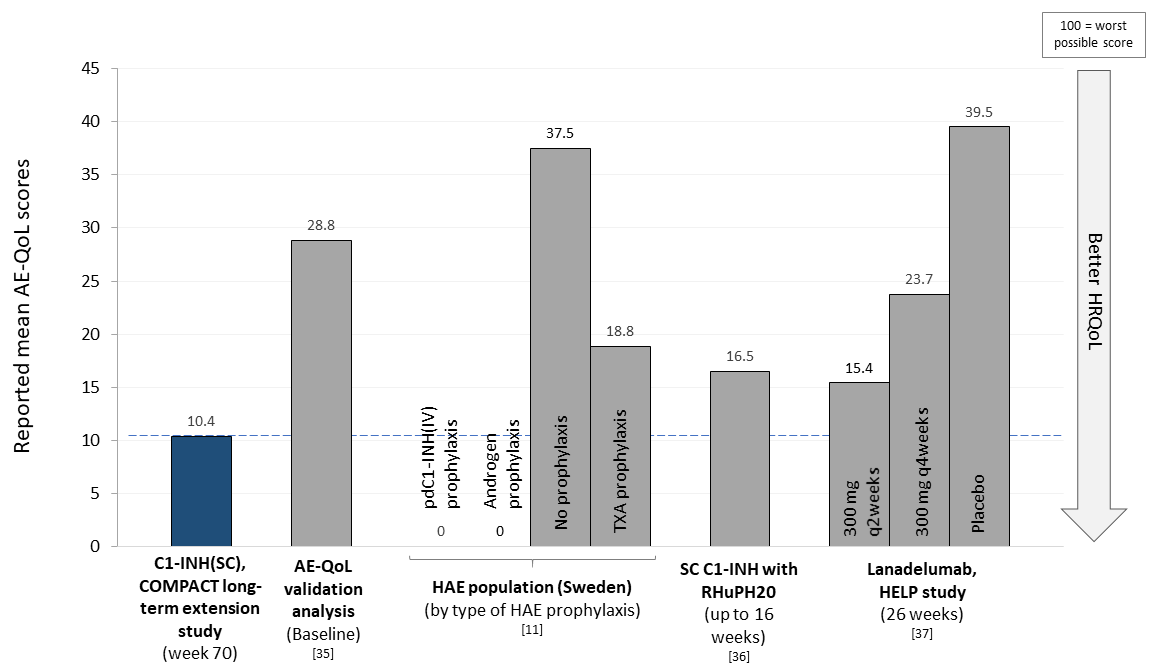


Note: The AE-QoL validation study included patients with recurrent angioedema due to chronic spontaneous urticaria as well as patients with hereditary angioedema.

C1-INH, C1-esterase inhibitor; C1-INH(SC), subcutaneous C1-inhibitor; pdC1-INH(IV), HRQoL, health-related quality of life; plasma-derived intravenous C1-INH
