# Supplementary material for: Long-term health-related quality of life in patients treated with subcutaneous C1-inhibitor replacement therapy for the prevention of hereditary angioedema attacks: findings from the COMPACT open-label extension study
Source: Orphanet J Rare Dis. 2021 Feb 15;16:86. doi: 10.1186/s13023-020-01658-4 (PMC7885603; doi:10.1186/s13023-020-01658-4)
Supplement: Supplementary file 7 — Additional file 7. Median AE-QoL and HAE-QoL score at different time points during the 88-week extension period, all C1-INH(SC) doses combined (US only). [file 13023_2020_1658_MOESM7_ESM.docx]

**Additional file 7**. Median AE-QoL and HAE-QoL score at different time-points during the 88-week extension period, all C1-INH(SC) doses combined (US only)^a^

| **AE-QoL Scores^b^**, median (25^th^, 75^th^ percentile)  (higher scores=greater impairment). | | | | |
| --- | --- | --- | --- | --- |
| Domain (range of possible scores) | Extension Week 6  (n=38) | Extension Week 22  (n=45) | Extension Week 46  (n=43) | Extension Week 70  (n=30) |
| Total Score (0-100) | 10.29  (4.41, 17.65) | 13.24  (5.88, 23.53) | 14.71  (2.94, 23.53) | 13.24  (2.94, 27.94) |
| Functioning (0-100) | 0  (0,0) | 0  (0,0) | 0  (0,0) | 0  (0,0) |
| Fatigue/Mood (0-100) | 17.50  (0, 30.00) | 25.00  (5.00, 40.00) | 20.00  (5.00, 40.00) | 20.00  (0, 40.00) |
| Fears/Shame (0-100) | 8.33  (0, 25.00) | 12.50  (4.17, 25.00) | 16.67  (0, 29.17) | 14.58  (0, 29.17) |
| Nutrition (0-100) | 0  (0, 12.50) | 0  (0, 12.50) | 0  (0, 12.50) | 0  (0, 12.50) |
|  |  |  |  |  |
| **HAE-QoL Scores^c^**, median (25^th^, 75^th^ percentile)  Global score range: 25 to 135 (higher scores=less impairment) | | | | |
| Domain (range of possible scores) | | Extension Week 26  (n=40) | Extension Week 50  (n=40) | Extension Week 74  (n=16) |
| Global Score (25-135) | | 126.0  (114.0, 133.5) | 128.0  (121.5, 133.0) | 128.5  (105.5, 132.5) |
| Physical Functioning and Health (4-23) | | 22.5  (20.0, 23.0) | 22.5  (20.5, 23.0) | 23.0  (20.5, 23.0) |
| Disease Related Stigma (3-15) | | 15.0  (14.0, 15,0) | 15.0  (13.0, 15.0) | 15.0  (13.5, 15.0) |
| Emotional Role and Social Functioning (4-20) | | 19.0  (18.0, 20.0) | 20.0  (18.0, 20.0) | 20.0  (17.0, 20.0) |
| Concern About Offspring (2-10) | | 9.0  (5.5, 10.0) | 10.0  (8.0, 10.0) | 9.0  (6.0, 10.0) |
| Perceived Control Over Illness (4-20) | | 18.0  (15.0, 20.0) | 19.0  (16.5, 20.0) | 19.0  (12.0, 19.0) |
| Mental Health (4-24) | | 23.0  (19.5, 24.0) | 23.0  (21.0, 24.0) | 22.0  (18.5, 24.0) |
| Treatment Difficulties (4-23) | | 23.0  (20.5, 23.0) | 22.5  (21.0, 23.0) | 21.5  (19.0, 23.0) |
| ^a^These assessments were not performed at baseline  ^b^The AE-QoL [31,35] is a validated angioedema-specific instrument comprised of 17 questions based on a recall period of 4 weeks. For the total and each domain, scores can range from 0 to 100 (higher scores indicate greater impairment).  ^c^The HAE-QoL [32] is a health-related QoL questionnaire specifically designed for studying the impact of HAE due to C1-INH(SC) deficiency on adult patients’ QoL. It consists of 25 items assigned to 7 dimensions (3 or 4 items per dimension); each item scored from 1 to 5 or 1 to 6. The global score reflects the sum of all 25 individual item scores (higher scores indicate better outcomes). | | | | |
