# Supplementary material for: Long-term health-related quality of life in patients treated with subcutaneous C1-inhibitor replacement therapy for the prevention of hereditary angioedema attacks: findings from the COMPACT open-label extension study
Source: Orphanet J Rare Dis. 2021 Feb 15;16:86. doi: 10.1186/s13023-020-01658-4 (PMC7885603; doi:10.1186/s13023-020-01658-4)
Supplement: Supplementary file 8 — Additional file 8. Mean HAE-QoL scores by individual domain along with comparative published data for the same domains in other HAE populations. [file 13023_2020_1658_MOESM8_ESM.docx]

**Additional file 8.** Mean HAE-QoL scores by individual domain along with comparative published data for the same domains in other HAE populations

A) Physical functioning
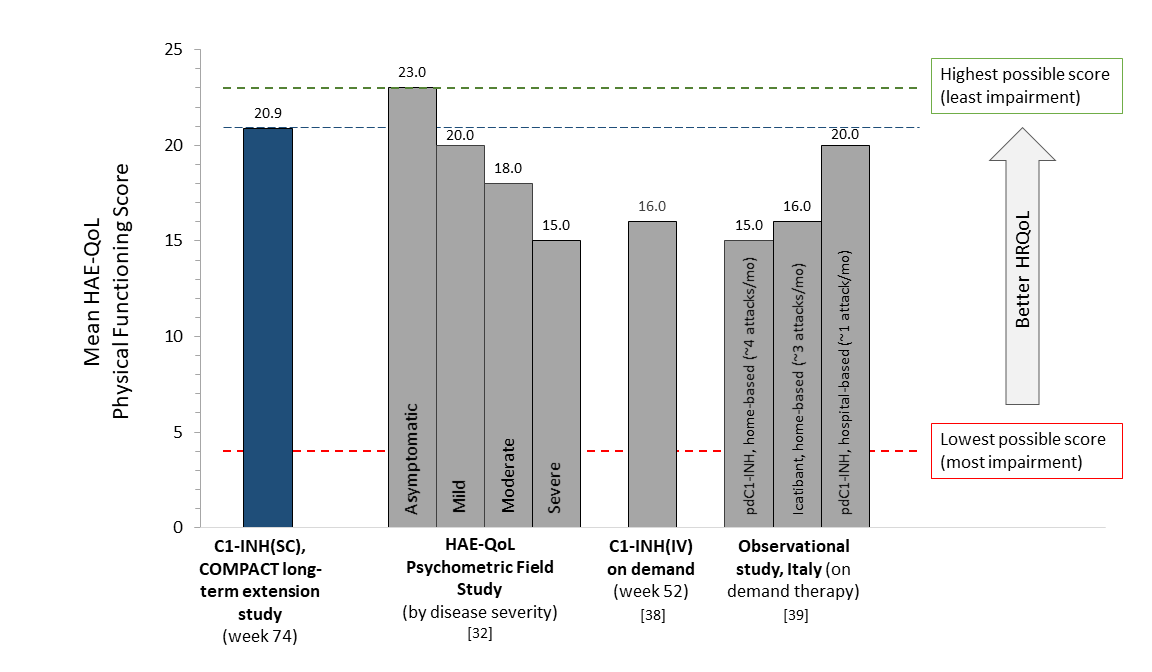


B) Disease-related stigma


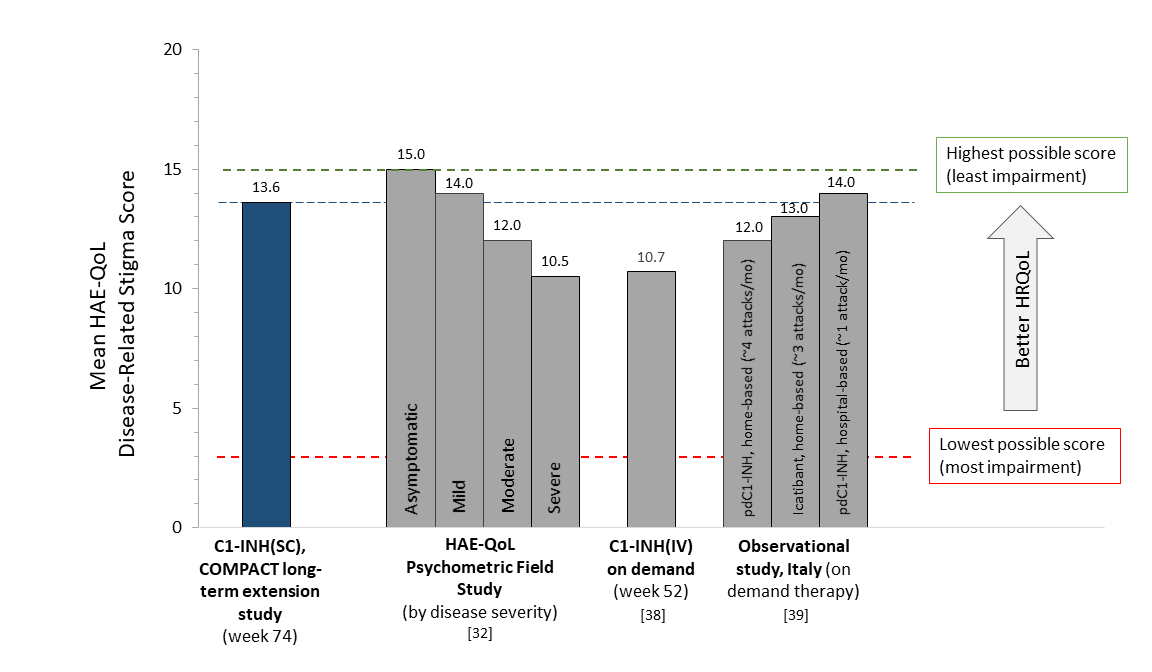


C) Emotional role and social functioning


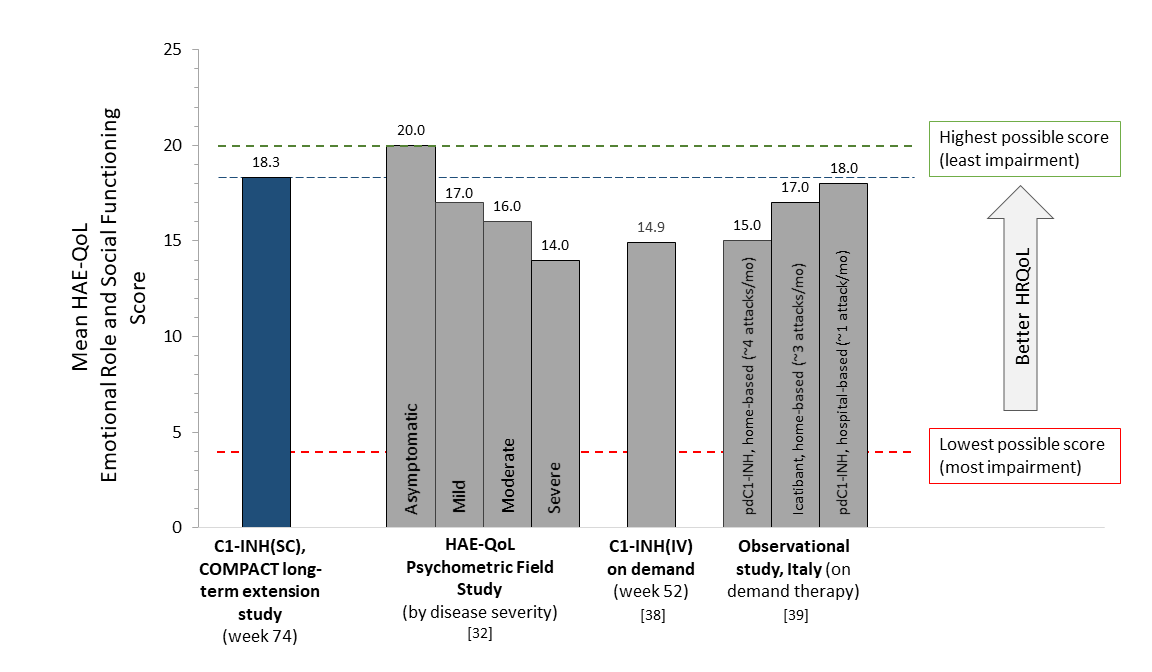


D) Concern about offspring


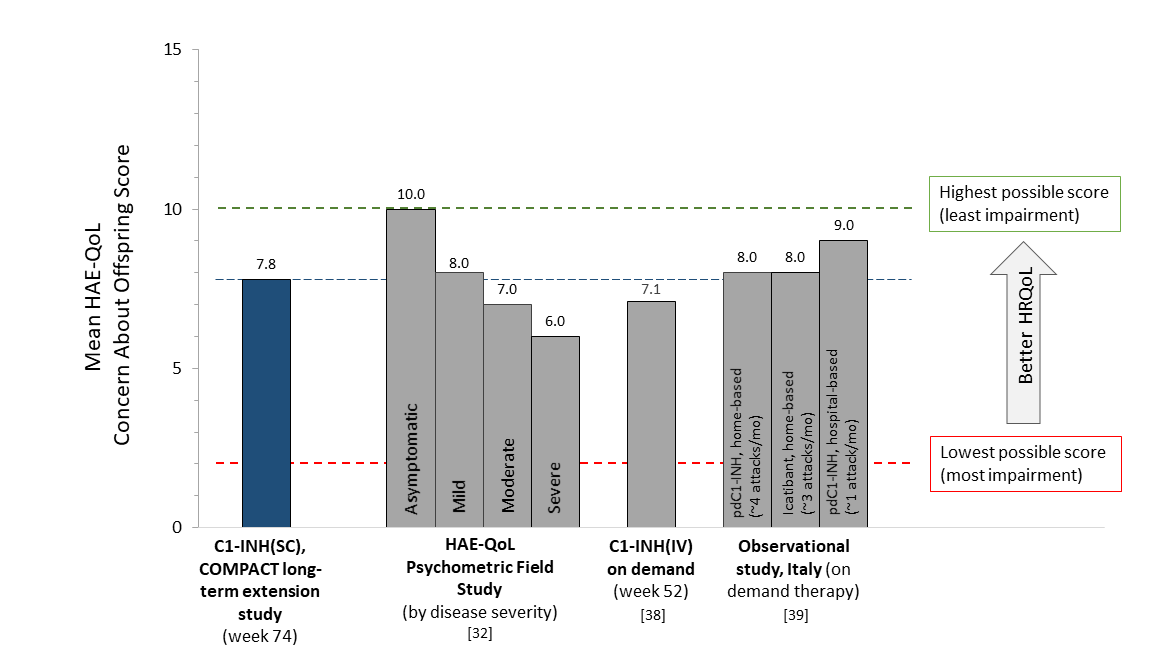


E) Perceived control over illness


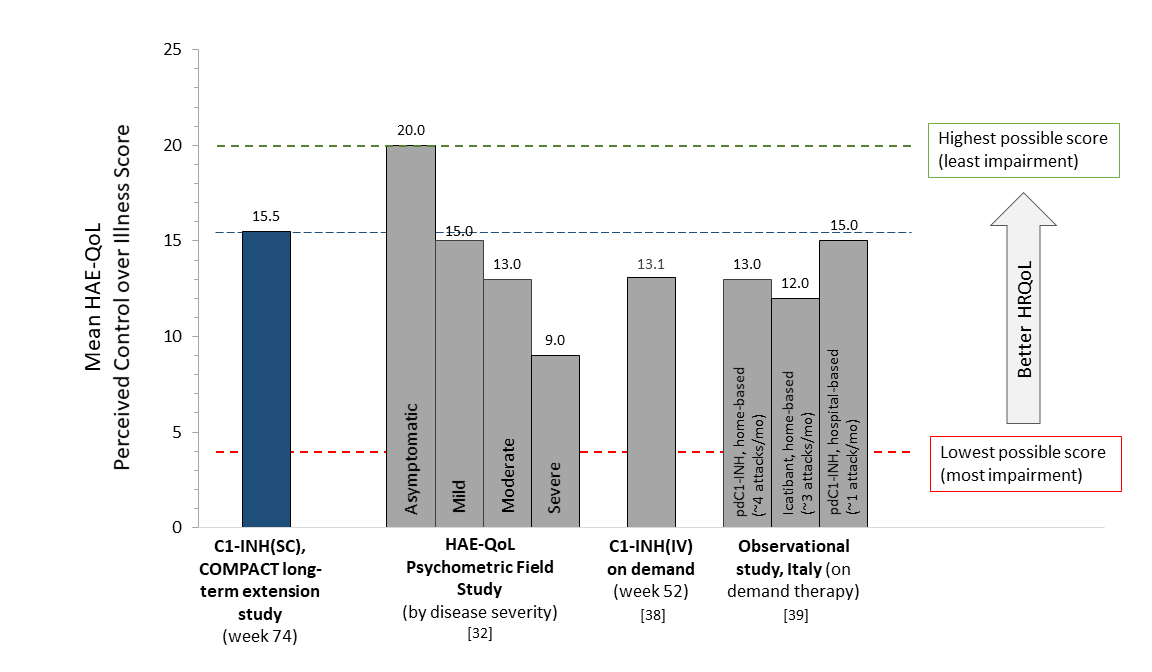


F) Mental health


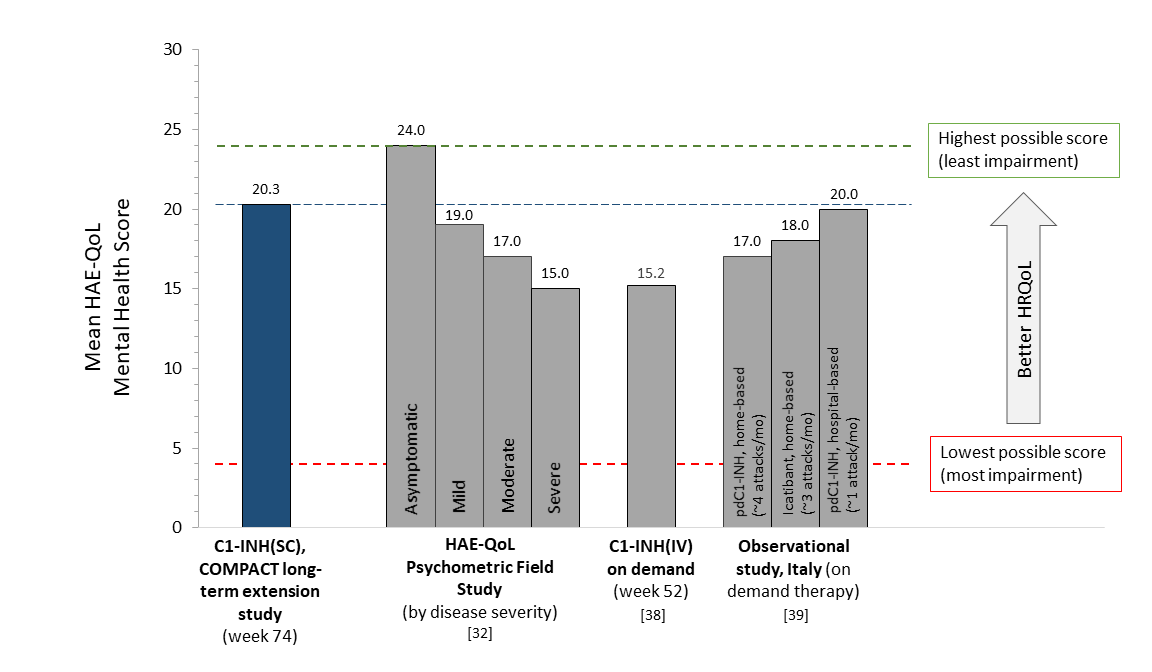


G) Treatment difficulties


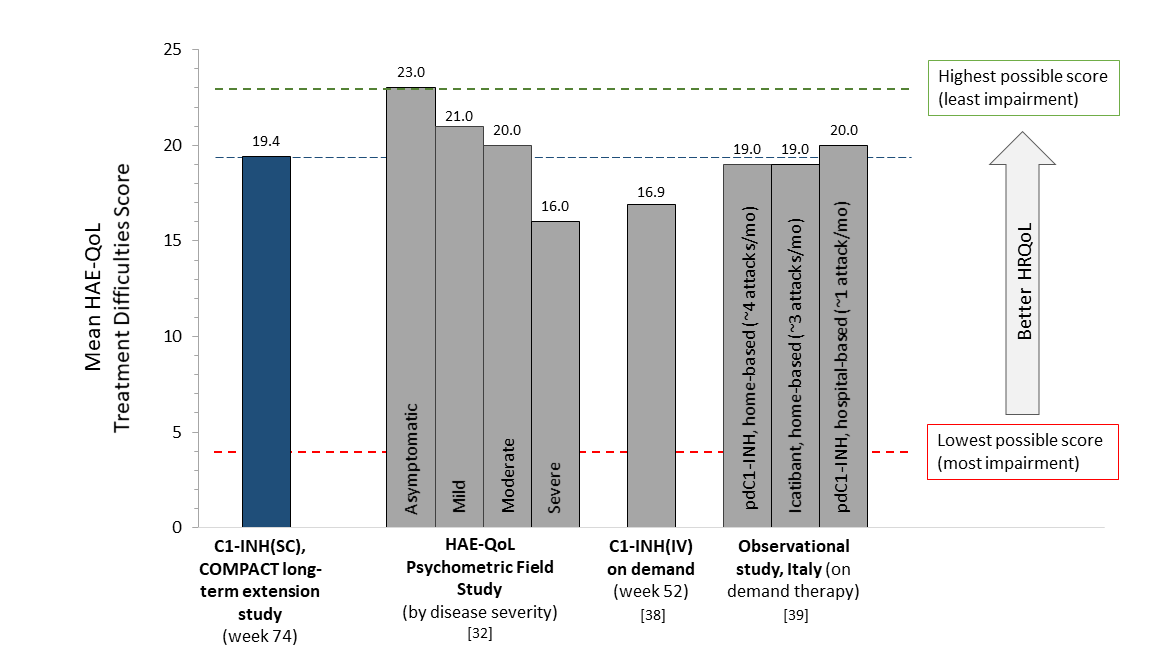


*Note*: Plot points for data from Squeglia 2016 study were extrapolated from a published figure

C1-INH(IV), intravenous C1-inhibitor; C1-INH(SC), subcutaneous C1-inhibitor; HRQoL, health-related quality of life pdC1-INH(IV), plasma-derived intravenous C1-IN
